# The Gene Encoding the RCC1 (Regulator of Chromosome Condensation 1) Protein in *Drosophila melanogaster* and *Homo sapiens*

**DOI:** 10.3390/ijms262311276

**Published:** 2025-11-21

**Authors:** Vera A. Turtapkina, Maria V. Maltseva, Elena V. Evtushenko, Sima S. Gatzkaya, Evgeniya S. Omelina, Nadezhda V. Battulina, Natalia A. Lemskaya, Victor V. Shloma, Alexander V. Vershinin, Tatyana Yu. Vatolina, Igor F. Zhimulev

**Affiliations:** Institute of Molecular and Cellular Biology, Novosibirsk 630090, Russia; v.turtapkina@g.nsu.ru (V.A.T.); maltseva@mcb.nsc.ru (M.V.M.); evt@mcb.nsc.ru (E.V.E.); jaits@mcb.nsc.ru (S.S.G.); battulina@mcb.nsc.ru (N.V.B.); lemnat@mcb.nsc.ru (N.A.L.); shloma@mcb.nsc.ru (V.V.S.); avershin@mcb.nsc.ru (A.V.V.); vatolina@mcb.nsc.ru (T.Y.V.)

**Keywords:** *RCC1* gene, cell cycle, RAN, *Drosophila melanogaster*, *Homo sapiens*, glioma, gene structure, developmental genes, housekeeping genes

## Abstract

The *RCC1* gene is active in ensuring many cellular functions related to cell division in *Drosophila melanogaster* and *Homo sapiens*. A detailed comparison of the structure and functions of the *RCC1* gene in *Drosophila melanogaster* and *Homo sapiens* was carried out using different analytical techniques (bioinformatics, immunofluorescence and confocal microscopy, FISH, and molecular genetic methods). The *Drosophila RCC1* gene belongs to the family of housekeeping genes, since it resides in the interbands and gray bands of polytene chromosomes within aquamarine/lazurite chromatin in *D. melanogaster*. Furthermore, the databases demonstrate that *RCC1* in *D. melanogaster* is expressed in all the tissues at all the developmental stages. According to The Human Protein Atlas, *RCC1* in humans also exhibits low tissue specificity for 29 tissues. Immunostaining of polytene chromosomes with RCC1 antibodies revealed approximately 260 sites of RCC1 protein localization exclusively in black bands (sites of developmental genes) and in heterochromatin. The size of the coding gene portions is almost identical for *D. melanogaster* and *H. sapiens*, being ~2 kb. The group of *Drosophila* proteins related to condensed chromatin, RCC1 being a member of this group, has homologs forming similar interaction networks in humans. The conserved nature of the *RCC1* gene has been confirmed by cell cycle studies in both species. It was found that expression of the *RCC1* gene is upregulated in glioblastoma; the *RCC1* protein predominantly resides on centrioles during metaphase.

## 1. Introduction

There is no doubt that *Drosophila* is currently the best-studied genetic object. Meanwhile, the research focus is gradually shifting toward humans. Therefore, comparative genetic studies are of significant interest. We compared the data for the *RCC1* (Regulator of Chromosome Condensation 1) gene and RCC1 protein in *Drosophila melanogaster* and *Homo sapiens*.

The RCC1 protein was identified approximately 40 years ago [1] as one of the key cell cycle regulators. It is the only known guanidine nucleotide exchange factor for nuclear Ras-like G protein (Ran) [2] and is directly involved in cellular processes in humans, such as nuclear envelope formation, nucleocytoplasmic transport, mitotic spindle assembly, and chromatin condensation during late S phase and early M phase of the cell cycle [3].

An analysis of the amino acid sequence of RCC1 protein in humans revealed that RCC1 consists of seven homologous repeats, each comprising 51–68 amino acid residues [4]. According to the NCBI database, it exists as three isoforms named RCC1-A, RCC1-B, and RCC1-C [5]. In human cells, RCC1 also exists as at least three isoforms referred to as αRCC1, βRCC1, and γRCC1, varying in their expression levels in certain tissues and differing in their affinity for chromatin and molecular interactions [6].

According to the Uniprot database, the human RCC1 protein has a molecular weight of 45 kDa [7], which is also consistent with experimentally obtained data [1]. For *Drosophila*, the molecular weight of the RCC1 protein is 59 kDa (according to Uniprot); however, various literature sources report an observed molecular weight of 68 kDa [8,9]. Biochemical analysis of the *Drosophila melanogaster* nuclear protein RCC1, identified using monoclonal antibodies, demonstrated that RCC1 is associated with nucleosomes and is released from chromatin by DNA-intercalating agents [8]. The sequence from amino acids 46 to 417, containing seven repeats of the first type, turned out to be highly evolutionarily conserved: 45% of amino acids in this region are conserved in seven similar tandem repeats of the human *RCC1* gene. The protein is localized in all bands on polytene chromosomes, without preference for any particular locus [8]. However, further studies revealed that RCC1 localizes only on black condensed bands and pericentromeric heterochromatin, so extensive regions of gray bands without RCC1 protein localization are observed on the chromosome. Such differential binding patterns of the same protein may indicate distinct activation mechanisms for developmental and housekeeping genes. Therefore, *RCC1* is a good marker for developmental genes localized within the black bands of chromosomes [10,11,12].

The RCC1 protein exists as two forms during the cell cycle: the soluble form and the chromatin-bound form. RCC1 distribution between these two states is regulated through its binding to RanBP1 and Ran to form a heterotrimeric complex [13]. As mentioned above, both *Homo sapiens* and *Drosophila melanogaster* have several RCC1 isoforms differing in affinity for chromatin and regulation mechanisms, suggesting that they have different functions during the cell cycle and in differentiated cells [6]. Recent studies have revealed that RCC1 plays an important role in tumorigenesis. Hence, *RCC1* expression is significantly downregulated in gastric cancer, and different *RCC1* expression suppression levels are observed [14]. However, *RCC1* expression during tumorigenesis is more likely to be upregulated compared to that in normal cells. It was demonstrated for such cancers as breast cancer, ovarian cancer, liver and pancreatic cancer [15], as well as clear cell renal carcinoma [16] lung adenocarcinoma [17] and colorectal cancer [18].

Gliomas are among the most malignant and difficult-to-treat types of cancer. Gliomas are a common and diverse group of neuroectodermal brain tumors [19]. Little data is available on changes in *RCC1* expression for this cancer type. Recent studies focusing on this topic have dealt with the interaction between arginine methyltransferase (protein arginine N-methyltransferase 6, PRMT6) and RCC1, leading to an increased concentration of RCC1 methylated at Arg214. In turn, it enhances its affinity for chromatin and activates Ran. In normal cells, this process is regulated through deubiquitylation and phosphorylation of PRMT6 by casein kinase 2 (CK2). Hence, disruptions in the CK2-PRMT6-RCC1 signaling axis result in upregulated *RCC1* expression [20].

To summarize, since RCC1 acts as one of the key cell cycle regulators and controls Ran activation, it is a promising target for studying its role in the development of various tumors, including gliomas. However, although many studies regard RCC1 as an onco-marker, differences in *RCC1* expression in gliomas and control tissue samples remain inconsistent across various sources [20,21].

The features of localization of the *RCC1* gene and its product in *D. melanogaster* and *H. sapiens* were identified in this work. The interactions of the RCC1 protein were analyzed. Additionally, a comprehensive comparative analysis of the cell cycle phases in *D. melanogaster* cells and in normal and cancer (glioma) human cells were performed.

## 2. Results and Discussion

### 2.1. The Structure of the RCC1 Gene and Localization of the RCC1 Protein in Polytene Chromosomes of D. melanogaster

**Figure 1:**
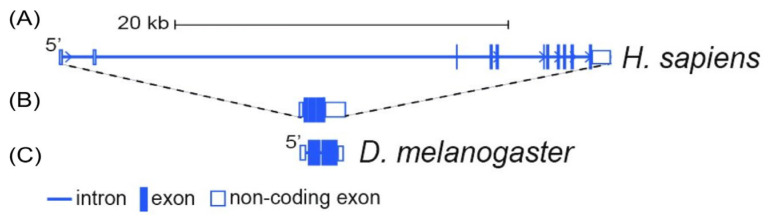
The structure and sizes of the *RCC1* gene according to the UCSC genome browser. (**A**) *Homo sapiens* (Genome version T2T CHM13v2.0/hs1); (**B**) the exon structure of the *RCC1* gene in *Homo sapiens*; (**C**) *Drosophila melanogaster* (Genome version BDGP Release 6 + ISO1MT/dm6).

The *RCC1* gene in humans was discovered by M. Ohtsubo et al. in 1989 [1]. The structure of the *RCC1* gene is well studied. The *Drosophila melanogaster* and *Homo sapiens* genes were compared using databases. Figure 1 shows the structure of the *RCC1* gene in both *Homo sapiens* and *Drosophila melanogaster*; Table 1 provides a comparative analysis of the structural organization of the gene across these two species.

The *RCC1* gene in *Homo sapiens* is approximately 14-fold larger than that in *Drosophila melanogaster* due to insertion of extensive introns between short exons. Contrariwise, in *Drosophila melanogaster*, long exons are separated by short intronic sequences. Therefore, genes have comparable lengths when only the exonic sequences are considered (Figure 1). Alignment of their nucleotide sequences (*RCC1 H. sapiens*: NM_001381865.2, *RCC1 D. melanogaster*: NM_079219.3) revealed 32% exon homology (data not shown).

The presence of polytene chromosomes in *D. melanogaster* offers a unique opportunity to localize the *RCC1* gene in a certain structure of the interphase genome. Previously, we identified four chromatin states by analyzing the distribution of proteins and histone modifications: aquamarine and lazurite chromatin, where housekeeping (HK) genes localize (promoters in aquamarine chromatin are interbands of polytene chromosomes, while gene bodies in lazurite chromatin are located in gray bands). The developmental genes are located in ruby chromatin (black bands and heterochromatin). Intergenic spacers and long introns are enriched in malachite chromatin [11,12].

**Figure 2:**
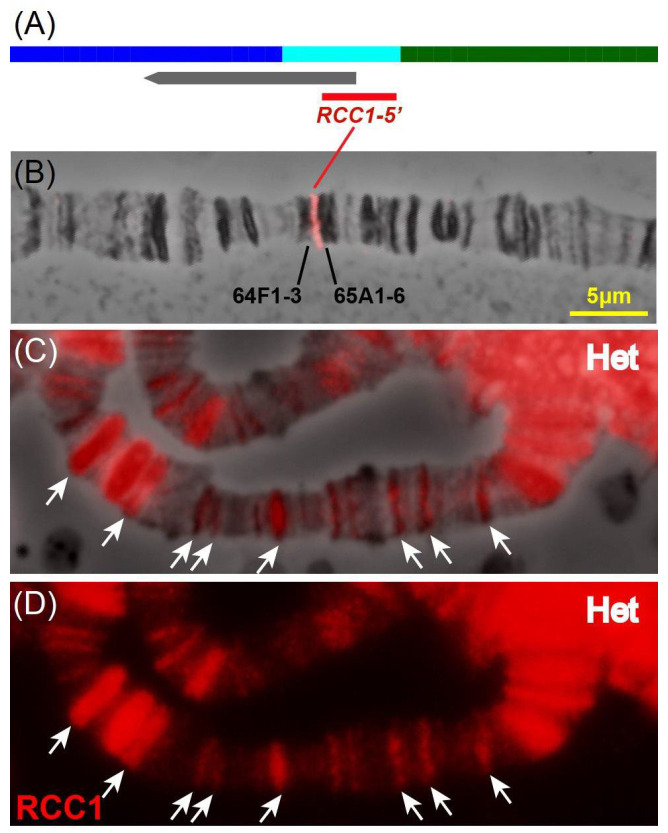
Mapping of the *RCC1* gene on the maps of chromatin states (**A**), polytene chromosomes (red signal in **B**), and RCC1 protein in black bands and pericentromeric heterochromatin of polytene chromosomes (**C**,**D**). (**A**) The 4HMM-predicted map of chromatin states [9,10] (top band), the *RCC1* gene (a gray arrow), and position of the FISH probe (the red fragment). (**B**) FISH localization of the 5′ fragment of the *RCC1* gene within the 64/65 3L region of *D. melanogaster* chromosome. (**C**,**D**) White arrows show localization of anti-RCC1 antibodies in black bands. Heterochromatin of polytene chromosomes named as Het (**C**—antibodies and phase contrast), (**D**—immunofluorescence only).

An analysis of Figure 2 demonstrates that the DNA probe from the 5′-end of the *RCC1* gene is localized in aquamarine chromatin, being localized in interbands in chromosomes, thus indicating that this gene belongs to the group of housekeeping genes [9,10] constituting ~50% of protein-coding genes (6562) in the *D. melanogaster* genome.

According to the FlyBase (FlyAtlas, modENCODE) database [22], *RCC1* in *D. melanogaster* is expressed in all the tissues at all the developmental stages. According to the Human Protein Atlas database, in *H. sapiens*, *RCC1* is also characterized by low tissue specificity for 29 tissues [21].

However, the gene product (RCC1 protein in the specified chromosomal region (C and D) localizes in inactive chromatin and predominantly in black bands where the developmental genes reside (3162 developmental genes in the *D. melanogaster* genome) or in heterochromatin.

**Figure 3:**
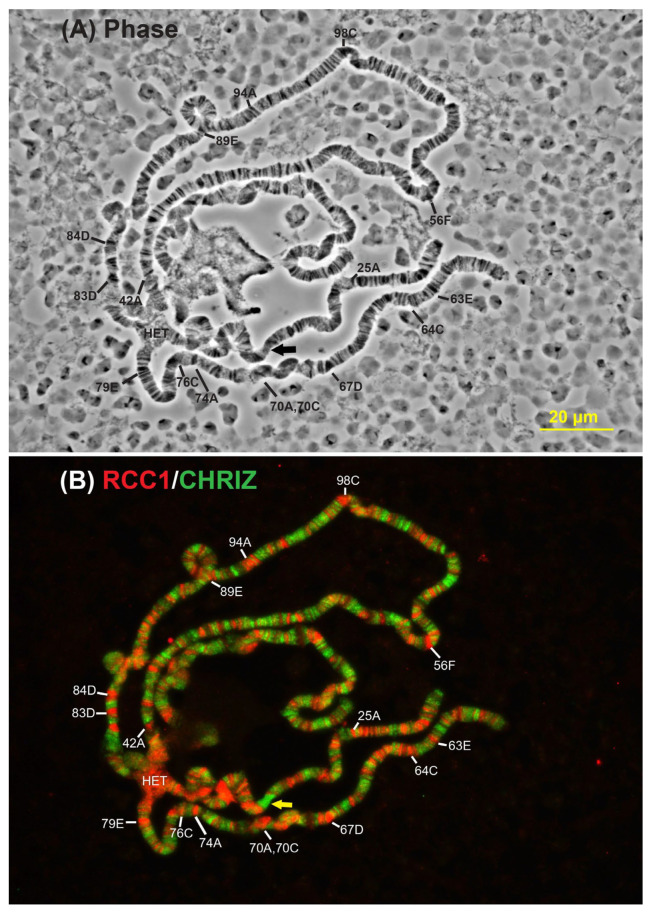
Cytological localization of RCC1 and Chriz on polytene chromosomes of the wild-type *Oregon-R* strain of *Drosophila melanogaster*. Regions corresponding to black bands are indicated by numbers and letters. An extended Chriz localization region is marked with a yellow arrow. The arrow shows the long tract of CHRIZ protein localization. HET denotes the pericentromeric heterochromatin.

Precise positions of the sites of labeling with anti-RCC1 antibodies in the full set of chromosomes were determined in wild-type and mutant *D. melanogaster* lines ([10], Table S1 and Figure 3).

Figure 3 shows the colocalization of antibodies targeting proteins specific to housekeeping genes and Chriz between black bands and antibodies targeting the *RCC1* gene product on *Drosophila melanogaster* polytene chromosomes of the wide-type *Oregon-R* strain.

Figure 3 shows the localization of antibodies targeting proteins Chriz specific to housekeeping genes and antibodies targeting the *RCC1* gene product on *Drosophila melanogaster* polytene chromosomes. It is worth mentioning that antibodies targeting the *Xenopus* RCC1 protein, which specifically bind to RCC1 proteins in both the *Drosophila melanogaster* and *Homo sapiens*, were used for immunostaining. Both products exhibit specific distribution patterns.

The extensive Chriz localization regions and numerous shorter regions are alternating with the areas stained positively for RCC1. One can see that RCC1 localizes in the pericentromeric chromatin regions as well as in the regions of black bands within the euchromatic arms of chromosomes. All the signals are exclusively localized to these regions. In other words, the RCC1 protein resides on chromosomal segments containing developmental genes [11]. A total of about 260 localization sites were identified for the *Rif1^1^* strain. The comprehensive list is provided in Table S1 (Supplementary Materials). The RCC1 signals were identical in all the studied strains (Figure 3, Table S1, [10]).

### 2.2. Characterization of RCC1 Protein

**Figure 4:**
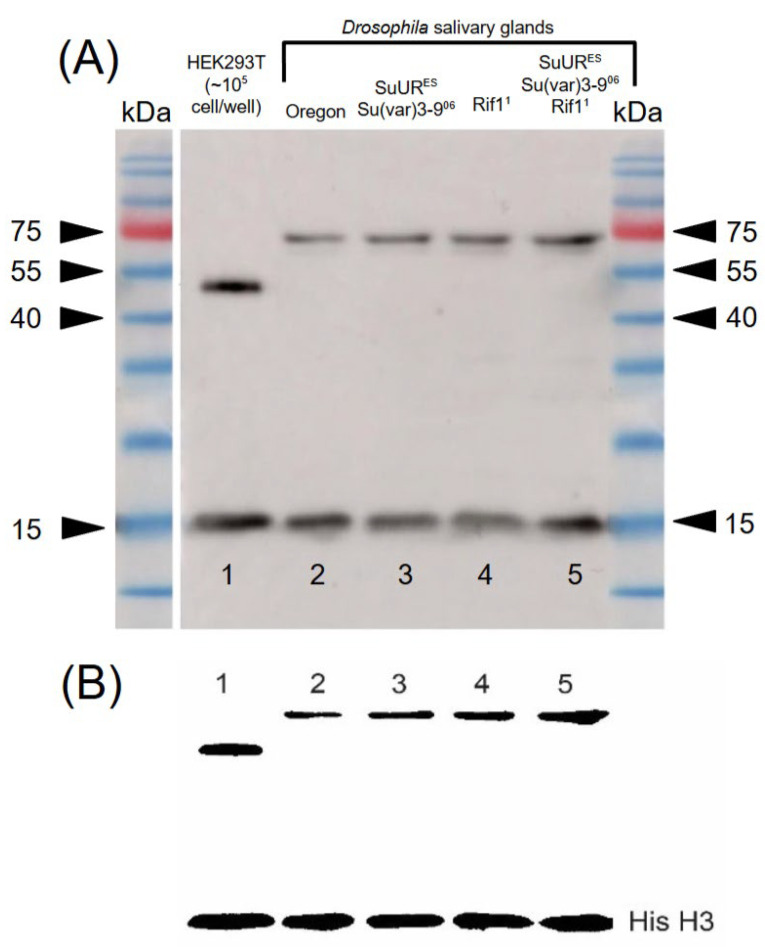
(**A**) Characterization of the RCC1 protein in *Drosophila melanogaster* and *Homo sapiens* by Western blot analysis: (rabbit polyclonal antibodies against *Xenopus* RCC1 protein 1:1000). (1) Human embryonic kidney cell culture (HEK 293T); (2) salivary glands of the *Oregon-R* strain of *Drosophila melanogaster*; (3) salivary glands of the *SuUR^ES^ Su(var)3-9^06^* strain of *Drosophila melanogaster*; (4) salivary glands of the *Rif1^1^* strain of *Drosophila melanogaster*; and (5) salivary glands of the *SuUR^ES^ Su(var)3-9^06^*, *Rif1^1^* strain of *Drosophila melanogaster*. (**B**) Analysis of the Western blot data using the ImageJ software; designations for lanes (1–5) are identical to those used in Figure 2A. The lower band at 15 kDa is a marker of lane loading and molecular weight—histone H3 (mouse monoclonal antibodies against histone H3—1:10,000). According to the manufacturer’s specification, the HEK239T cell line is a positive control for anti-*Xenopus* RCC1 antibodies.

In 1991, M. Frasch identified a *Drosophila* homolog of the *RCC1* gene, which was named *BJ1*. Using monoclonal antibodies, M. Frasch identified this nuclear protein and mapped it to polytene chromosomes; it was found to be associated with bands, without preference for specific loci. However, as we now know, there are two types of these bands: black bands containing developmental genes and gray bands, which contain housekeeping genes, together with interbands. These gene groups differ both at the genetic (nucleotide sequence) and the epigenetic (the level not involving changes to the DNA structure) levels. The genomic localization of the RCC1 protein in the *Drosophila melanogaster* genome was previously discussed in report [10]. The present study provides more detailed localization data obtained for two strains: *Rif1^1^* and *SuUR^ES^*, *Rif1^1^*, *Su(var)3-9^06^*. In our study, we revealed that antibodies targeting *Xenopus* RCC1 protein specifically bind to the RCC1 protein of both *Drosophila melanogaster* and *Homo sapiens*, as demonstrated by Western blot analysis (Figure 4).

Black bands and the chromocenter are known to frequently undergo incomplete polytenization (under-replication), resulting in underrepresentation of the material from black bands in wild-type chromosomes. We employed Western blot hybridization to determine the molecular weight and perform a semi-quantitative analysis of the RCC1 protein in the Oregon-R strain of *Drosophila melanogaster* and in strains carrying different doses of mutant genes that restore under-replicated DNA regions in *Drosophila* chromosomes (Figure 3). It was demonstrated using the ImageJ software that in the mutant strains *SuUR^ES^ Su(var)3-9^06^*, *Rif1^1^*, and *SuUR^ES^ Su(var)3-9^06^ Rif1^1^*, the relative content of RCC1 protein in salivary gland cells increases more than threefold compared to the wild-type strain (Figure 4A,B).

Under-replication in polytene chromosomes of *Drosophila melanogaster* can be significant (up to 75% of DNA under-replication in black bands and pericentromeric heterochromatin). Mutations in *SuUR^ES^* or *Rif1^1^* proteins suppress under-replication and repair the target amount of DNA per individual chromatid [23].

Therefore, the data shown in Figure 4B allow one to infer that, indeed, the amount of DNA is restored, and the quantity of material in black bands and chromocenter increases. Since RCC1 binds to these structures, the quantity of RCC1 protein also rises. This finding proves once again that the antibodies used are specific to under-replicated chromosomal regions (i.e., RCC1 binding sites).

**Figure 5:**
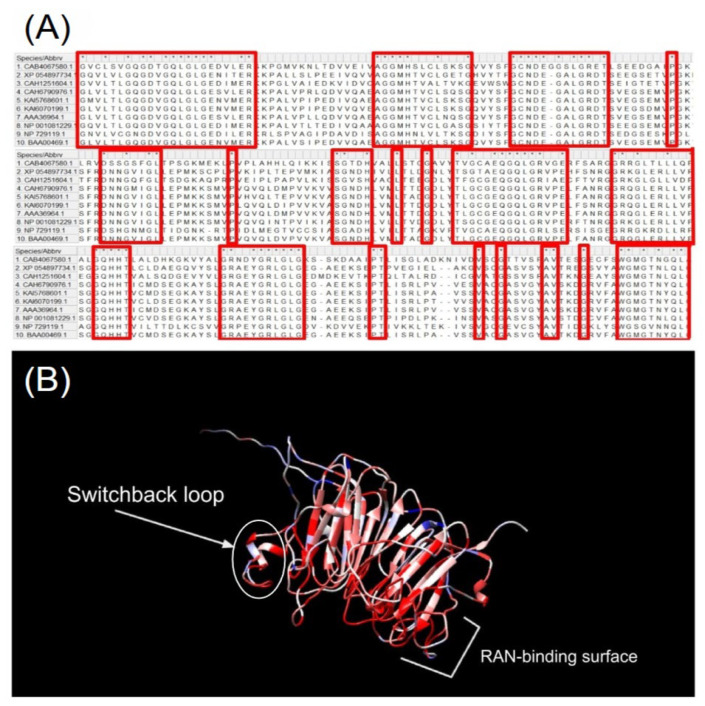
(**A**) Sequence alignment regions of the RCC1 protein for ten different organisms: *Homo sapiens* (BAA00469.1), *Drosophila melanogaster* (NP_729119.1), *Xenopus laevis* (NP_001081229.1), *Cricetus cricetus* (AAA36964.1), *Marmota monax* (KAI6070199.1), *Gulo gulo* (KAI5768601.1), *Phodopus roborovskii* (CAH6790976.1), *Branchiostoma lanceolatum* (CAH1251604.1), *Poeciliopsis prolifica* (XP_054897734.1), and *Lepeophtheirus salmonis* (CAB4067580.1). Homologous regions are shown in red, * show identical amino acids. (**B**) The model of human RCC1 protein (PDB id 1I2M), with the conservatism map plotted using the Chimera 1.14 software. The most conserved regions are shown in red; the least conserved ones are shown in blue. The oval in Figure 5B denotes the switchback loop. The RAN-binding surface is shown with a bracket.

Unlike the *RCC1* gene, RCC1 protein is more conserved, human RCC1 protein has many homologs among various organisms. Figure 5 shows (A) the RCC1 protein sequence alignment regions in ten organisms and (B) the model of human RCC1 protein, with the conservatism map built for these organisms.

**Figure 6:**
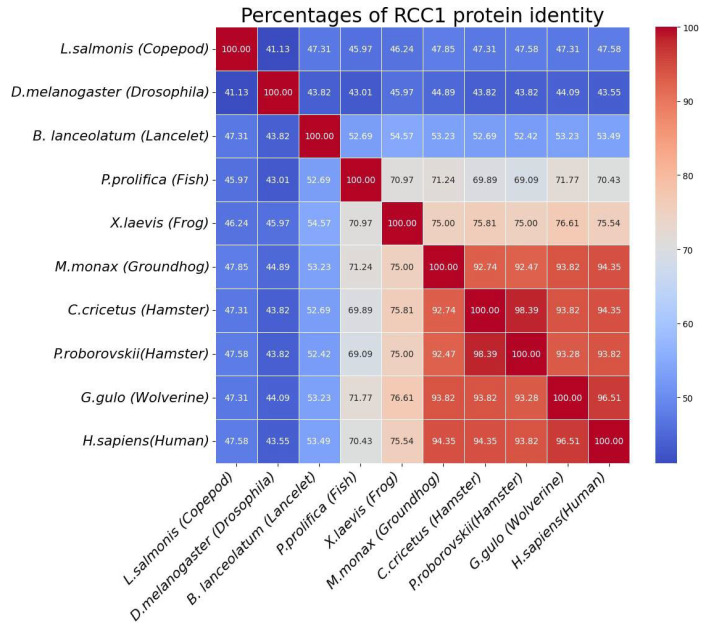
The homology percentage of amino acid sequences of RCC1 protein for ten different organisms available in the NCBI database. The blue-to-red gradient indicates an increasing percentage of protein identity.

Despite the small percentage of the total homology between some organisms, certain regions in RCC1 protein still remain intact. The region responsible for RCC1–RAN binding and the switchback loop required for binding to nucleosome remain especially conserved [24]. Figure 6 shows detailed information on homology percentage in these organisms.

**Figure 7:**
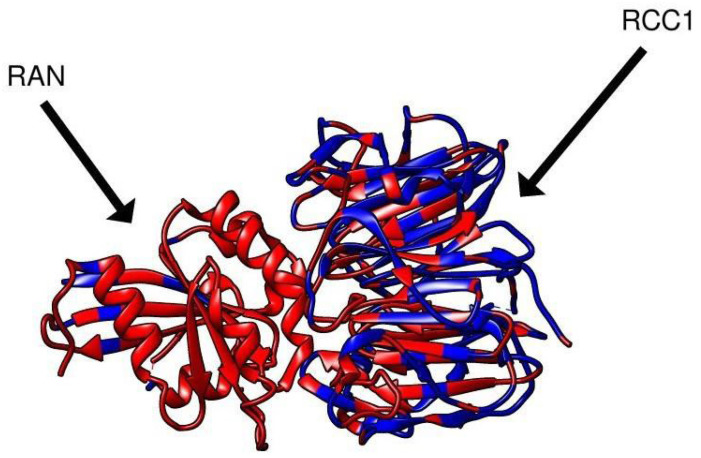
The model of RCC1–RAN interactions in *Homo Sapiens* (PDB id 1I2M), with the conservatism map between *Homo Sapiens* and *Drosophila melanogaster* additionally plotted using the Chimera.1.14 software. Conserved regions are shown in red; the differing regions are shown in blue.

The model showing the conserved regions of RCC1 and RAN for *Drosophila* 289 *melanogaster* and *Homo sapiens* is shown individually in Figure 7. The model of RCC1–RAN interaction, with the conservatism map between *Drosophila melanogaster* and *Homo sapiens*, is presented.

The model demonstrates that despite the large total percentage of blue color, the switchback loop and the RAN-binding surfaces are conserved.

**Figure 8:**
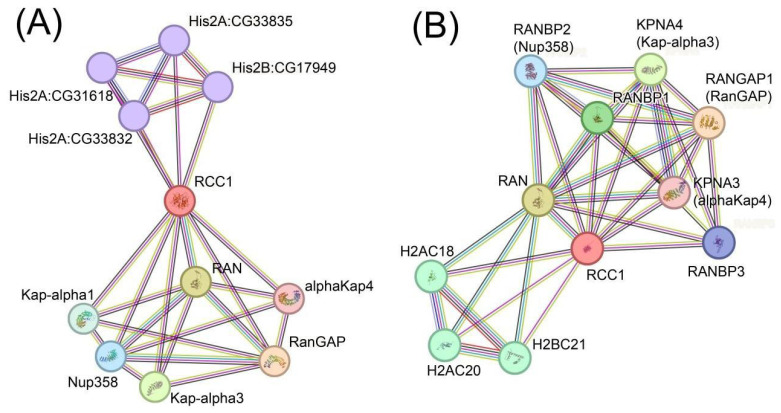
(**A**) Top ten interactions for the RCC1 protein in *Drosophila melanogaster* predicted according to the STRING database. (**B**) Top ten interactions for the RCC1 protein in *Homo sapiens* predicted according to the STRING database. The colors of lines denote different criteria for predicting the interaction: aquamarine—the interaction is specified in both databases; fuchsia—the interaction was verified experimentally; green—genetic neighborhood; red—gene fusion, blue—genetic coincidence; black—co-expression; light purple—protein homology; and light green—interaction is predicted according to the literature data. Homologous proteins are shown with circles of identical color.

When further studying the interactions of RCC1 protein in *Drosophila melanogaster* and *Homo Sapiens*, we placed a closer focus on proteins predicted to interact with RCC1 in both species. Ten proteins for each of the species, predicted to interact with RCC1 according to the STRING database [25], are presented in Figure 8.

Figure 8 indicates that both in *Homo sapiens* and *Drosophila melanogaster*, the RCC1 protein interacts with histone proteins, Ran and Ran-related proteins, as well as importins. Thus, Kap-alpha1 is a homolog of KPNA6; alphaKap3, a homolog of KPNA4/KPNA3; alphaKap4, a homolog of KPNA3/KPNA4; RanGAP, a homolog of RanGAP1; and Nup358, a homolog of RanBP2.

**Figure 9:**
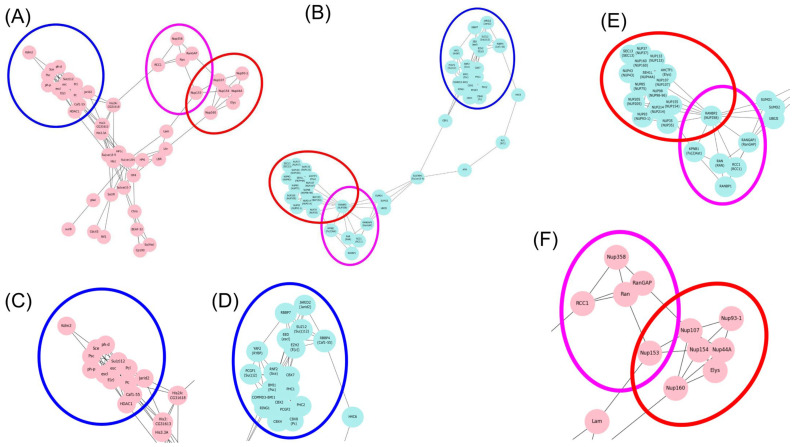
Non-directed interaction graphs for heterochromatin proteins in *D. melanogaster* (**A**,**C**,**F**) and homologous groups of *Homo sapiens* proteins (**B**,**D**,**E**) according to the STRING database. Functionally similar groups predominantly consisting of homologous proteins for *Homo sapiens* and *Drosophila melanogaster* are enclosed in ovals of identical colors (blue—for the Polycomb group; red—for the nucleoporin group; purple—for RAN and RAN-related proteins).

Polytene chromosomes in *Drosophila melanogaster* enable detailed analysis of the localization of various proteins in the genome, which is infeasible for *Homo sapiens* chromosomes. Taking into account the predominant localization of RCC1 within densely packed DNA regions (heterochromatin and black bands) (Figure 3), proteins associated with heterochromatin and black bands in *D. Melanogaster* were identified: aurB, D1, eff, Elys, E(z), His1, HP4, Lam, HP3, Nup93-1, Pc, Pcl, Psc, Sce, Ran, Rap1, RCC1,Su(var)3-9, Su(var)205, HP1a, su(Hw), and SuUR [26,27,28,29,30,31,32,33,34,35,36,37,38,39]. Proteins interacting with them were retrieved, and repeats for several proteins were identified. The STRING database used for predicting interaction employs the following criteria: the interaction is indicated in other databases; the interaction has been verified experimentally; genetic neighborhood; gene fusion; genetic coincidence; co-expression; protein homology; and frequent mentioning of co-occurring genes in the literature. Each predicted interacting protein was then compared to the original list of heterochromatin-associated proteins. An undirected graph of interactions (Figure 9A) was plotted using the Network X library for Python 3.10.12 as a result of this analysis.

Figure 9A indicates that in *D. melanogaster*, proteins were divided into several groups having a common function. The function was analyzed using the DAVID bioinformatics tool and the Uniprot database. Table 2 shows the distribution of genes with respect to their functions. Next, proteins homologous for *Homo sapiens* were retrieved for these proteins using the FlyBase database [22], and identical manipulations were performed for them. An undirected graph of interactions (Figure 9B) was plotted as a result of analysis. Figure 9B demonstrates that human proteins were also classified into groups according to the common function. Table 3 lists classification of proteins according to their functions.

Interestingly, according to recent data, the NUP98 and SEC13 proteins may also be involved in heterochromatin repair [40].

The results of this analysis demonstrate that the selected group of *D. melanogaster* proteins bound to condensed chromatin has homologs forming similar interaction networks among *H. sapiens* proteins. Hence, the results of modeling and cytological analysis are consistent and allow one to consider RCC1 as being localized within transcriptionally inhibited regions. Cytological data cannot be obtained for *Homo sapiens*; however, the modeling data are reproduced, with identical protein groups being singled out.

As it follows from the modeling data, RCC1 exhibits an affinity for Nucleoporin proteins (Nups) (Figure 9A,B,E,F). We analyzed the distribution of NUP93-1 in *D. melanogaster* nuclei and chromosomes.

**Figure 10:**
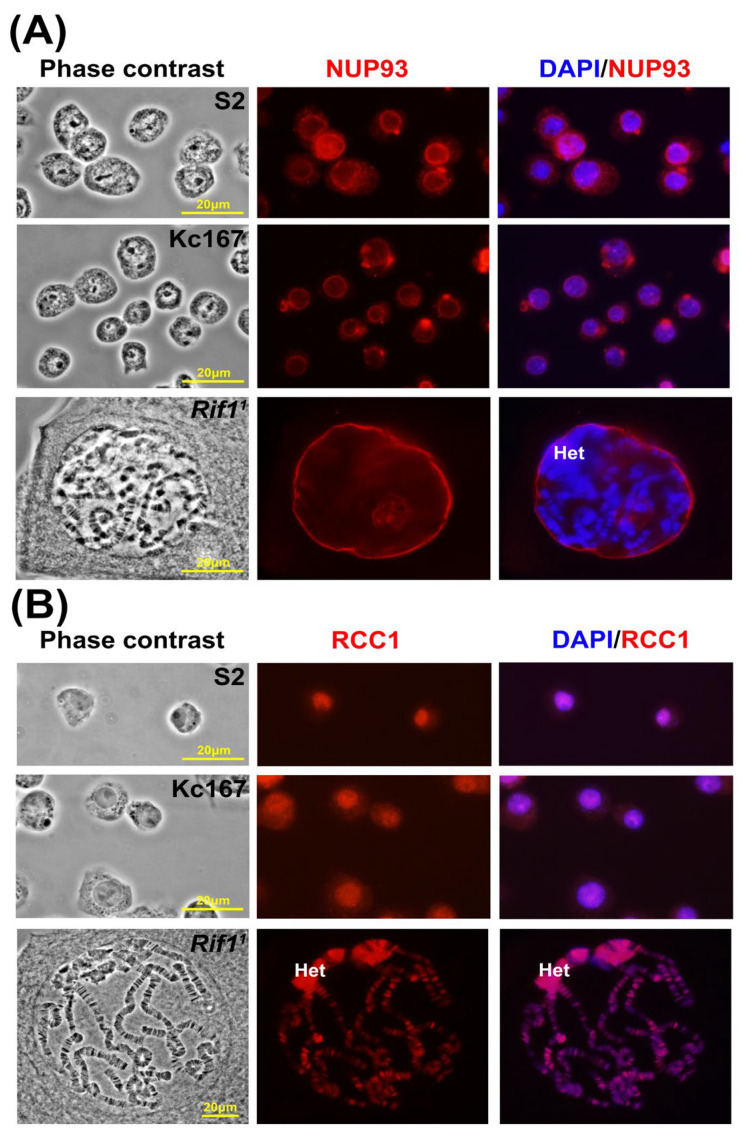
Localization of anti-NUP 93-1 (**A**) and anti-RCC1 (**B**) antibodies in interphase nuclei of cell cultures and unsquashed nuclei of *D. melanogaster* polytene chromosomes. (**A**) Top row: S2 cells; from left to right: phase contrast, NUP93-1 staining, superposition of NUP93-1 and DAPI; middle row: the same for Kc167 cells; bottom row: the same for nuclei with polytene chromosomes for *Rif1^1^* line. (**B**) The same as in (**A**) for RCC1 protein.

In interphase nuclei of cell cultures and unsquashed nuclei with polytene chromosomes of salivary glands, NUP93-1 binds exclusively to the nuclear envelope, and no labeling signs were identified in the bulk of the nucleus (Figure 10A). Meanwhile, anti-RCC1 antibodies easily penetrate into the unsquashed nucleus and stain chromosomes (Figure 10B).

The data obtained for localization of RCC1 and NUP93-1 in the *D. melanogaster* cell culture and unsquashed nuclei with polytene chromosomes explicitly contradict Figure 9, which indicates that RCC1 in *H. sapiens* and *D. melanogaster* belongs to groups of proteins having shared functions. In other words, RCC1 and NUP93-1 are supposed to interact in chromosomes both in *H. sapiens* and *D. melanogaster* as demonstrated by the 4HMM model according to which Lamin proteins within the nuclear envelope are expected to be localized in ruby chromatin.

Furthermore, it has been demonstrated recently that the component of the nuclear pore complex Nup93-1 is involved in repression mediated by Polycomb proteins, which are known to localize to black bands [23], and marks PRE-chromatin elements [35].

**Figure 11:**
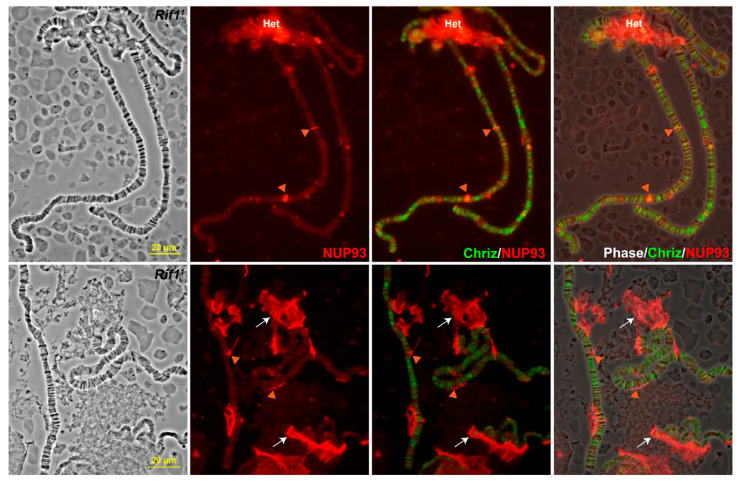
Localization of anti-NUP93 antibodies and CHRIZ on squashed specimens of salivary gland chromosomes. Red arrowheads indicate putative contacts with the nuclear envelope. White arrows indicate remnants of the nuclear envelope.

However, Figure 2 and Figure 3 indicate that in polytene chromosomes, anti-RCC1 antibodies intensively stain heterochromatin. One can see in Figure 11 that envelope fragments labeled with anti-NUP93-1 antibodies colocalize with pericentromeric heterochromatin (Het), which always localizes to the inner side of the nuclear envelope. Therefore, one can hypothesize that NUP93-1 and RCC1 actually have shared interactions. It can be indicated by individual signals from NUP93-1 (red arrow heads) shown in Figure 11, which can represent residual contacts between RCC1 and the nuclear envelope. These contradictions are partially resolved by the fact that in Figure 10B, the contacts are partial contacts between NUP93-1 proteins and pericentromeric heterochromatin, which almost always resides on the inner side of the nuclear envelope in the interphase nucleus.

Many additional studies both for *H. sapiens* and *D. melanogaster* need to be conducted to resolve this contradiction, and it harbors a potential for new discoveries.

### 2.3. RCC1 Localization Throughout the Cell Cycle

We used anti-xenopus RCC1 antibodies whose epitope resides in the conserved portion of the RCC1 protein (Figure 4), so the same antibodies could be used for immunostaining of all the RCC1 protein isoforms for both *D. melanogaster* and *H. sapiens*.

**Figure 12:**
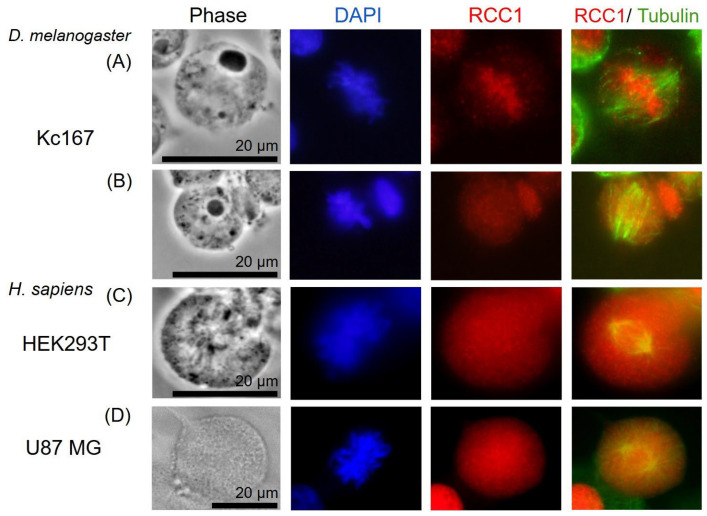
Optical microscopy images of the localization of RCC1 (red) and alphaTub (green) proteins during metaphase. Immunostaining of Kc167 cells (**A**,**B**) (*D. melanogaster*) and HEK293T (**C**), U87MG cells (**D**) (*H. sapiens*). Fixation with 3.7% formaldehyde.

**Figure 13:**
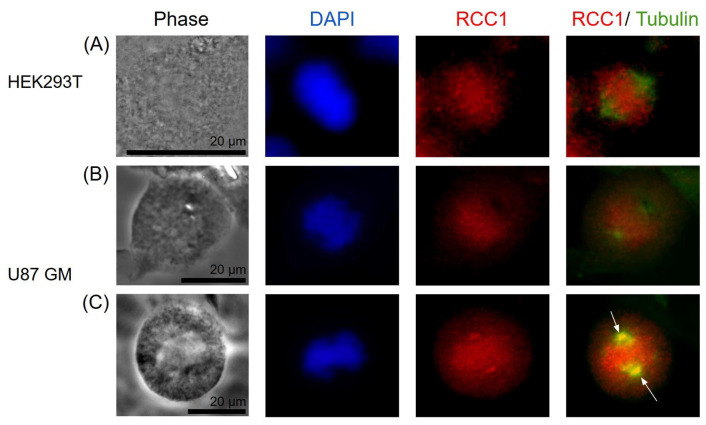
Optical microscopy images of the localization of RCC1 (red) and alphaTub (green) proteins during metaphase. Immunostaining of HEK293T (**A**) and U87MG cells (**B**,**C**) (*H. sapiens*). Acetone–methanol fixation. The white arrows indicate the accumulation of the RCC1 signal near the centrioles.

**Figure 14:**
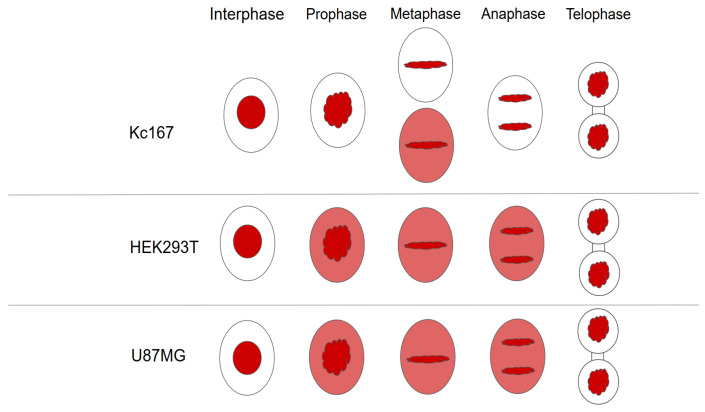
The scheme of RCC1 localization (shown in red) during the cell cycle for such cell lines as Kc167, HEK293T, and U87MG upon fixation with 3.8% formaldehyde.

**Figure 15:**
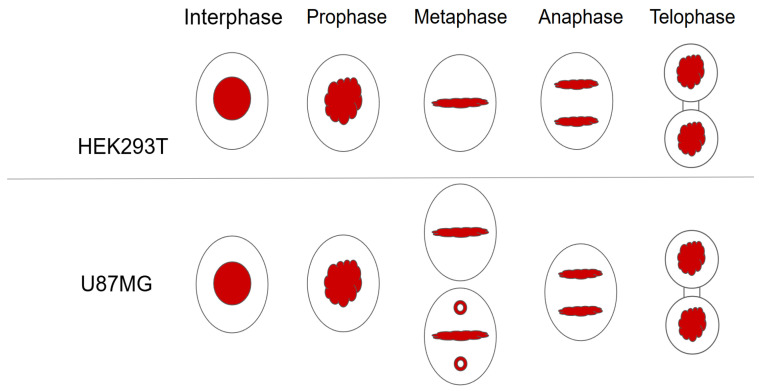
The scheme of RCC1 localization (shown in red) during the cell cycle for such cell lines as Kc167, HEK293T, and U87MG upon fixation with acetone and methanol.

RCC1 localization is known to be dependent on the fixation method [41]. It is generally believed that upon acetone–methanol fixation, RCC1 is detected on chromatin throughout the entire cell cycle, whereas when cells are fixed with formaldehyde, RCC1 colocalizes with chromatin exclusively during interphase and is not detected on metaphase chromosomes. Moore et al. expressed fusion with green fluorescent protein (GFP-RCC1) in human cells. Live transfected cell follow-up demonstrated that GFP-RCC1 resided in the nucleus during interphase and localized predominantly to chromatin at all stages of mitosis, although the signal dispersed in the cytoplasm was intensified during metaphase/anaphase [41]. In our study, we analyzed the RCC1 localization throughout the cell cycle using immunostaining with anti-RCC1 antibodies for two different fixation conditions. For formaldehyde fixation, which induces covalent interprotein cross-links, one can observe both the chromatin-bound and soluble forms of RCC1. Figure 12 and Figure 13 show the optical microscopy images of RCC1 localization during metaphase for different fixation methods. The general RCC1 localization in the cell cycle for Kc167 (*D. melanogaster*), U87 (*H. sapiens*), and HEK293T (*H. sapiens*) cell lines is shown in the scheme (Figure 14 and Figure 15); the remaining photographs are provided in Supplementary materials (Figures S1–S8, Tables S3 and S4).

Fixation with acetone and methanol could not be used for these antibodies for the Kc167 cell line (*D. melanogaster*). Therefore, Figure 13 shows only the human cell lines.

Figure 14 demonstrates that during interphase and telophase, RCC1 colocalized with chromatin in all the cell lines. During metaphase, the protein was distributed across the entire cell, being slightly concentrated within chromatin; for the *H. sapiens* cell lines, RCC1 was found to localize within alpha-tubulin. For *D. melanogaster* cells during metaphase, we observed both protein distribution across the entire cell and its colocalization with chromatin.

Our findings on localization of RCC1 protein for Kc167 and HEK293T cell lines agree with the known data [41]. However, RCC1 localization near microtubules within the spindle in metaphase is detected for the U87MG cell line upon fixation with acetone and methanol.

**Figure 16:**
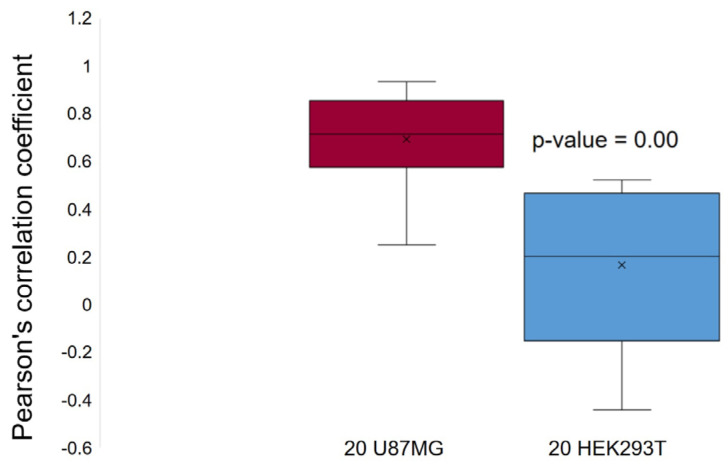
Pearson correlation coefficients (RCC1/Tub) for the HEK293T cell line and U87MG human glioblastoma cell line (optical microscopy images).

**Figure 17:**
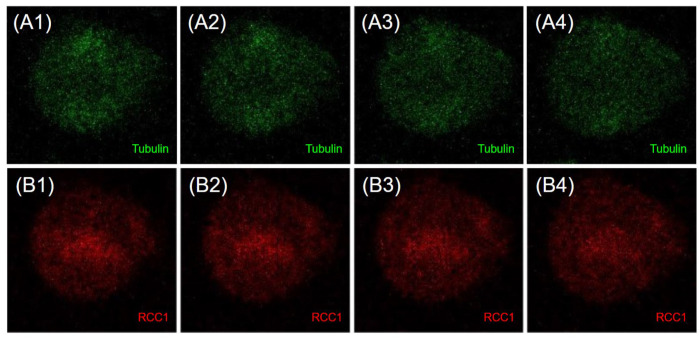
Confocal microscopy images of the localization of alphaTub protein (green) (**A1**–**A4**) and RCC1 (red) (**B1**–**B4**) during metaphase Immunostaining of Kc167 cells (*D. melanogaster*). Fixation with 3.7%formaldehyde.

**Figure 18:**
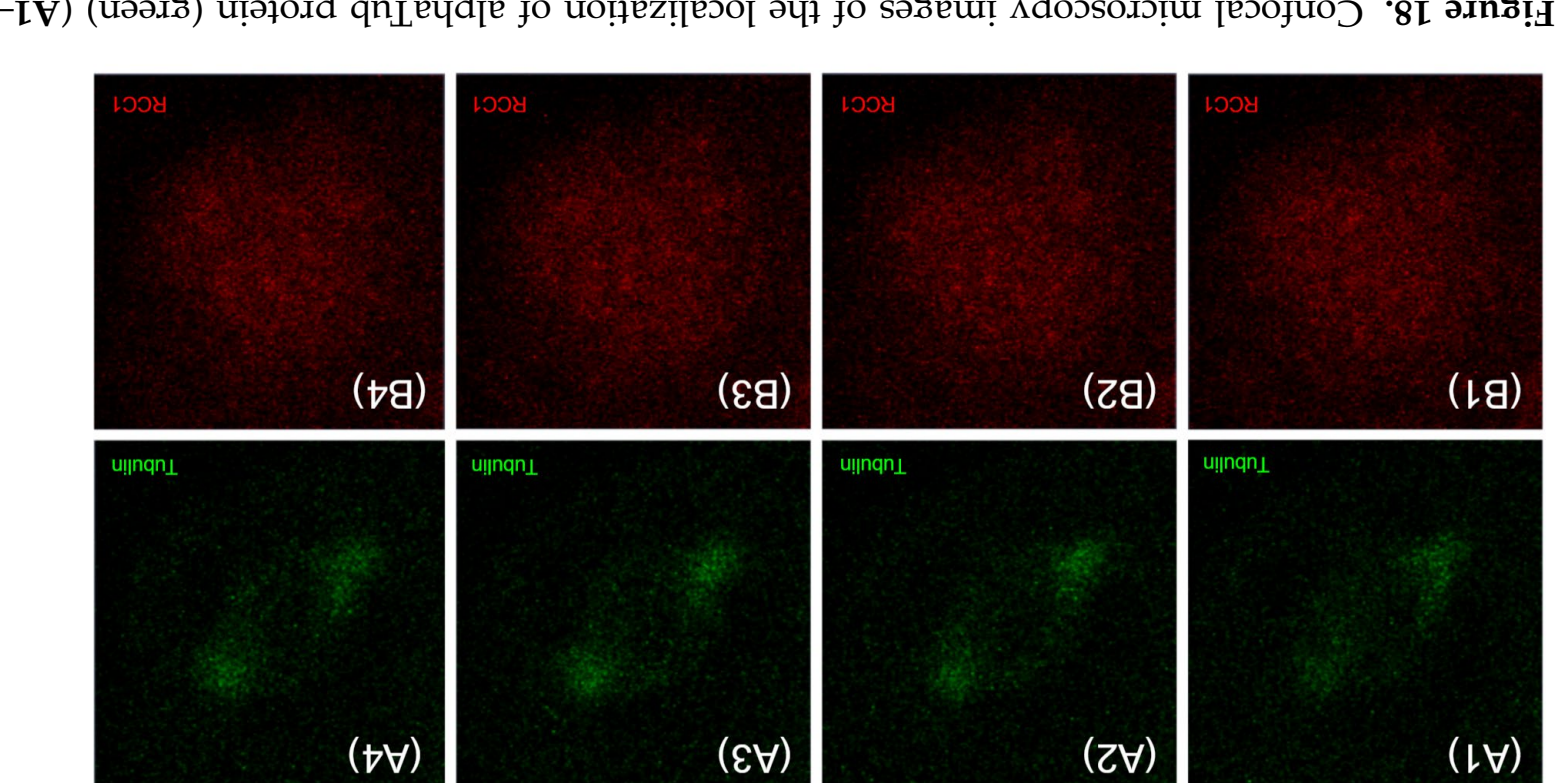
Confocal microscopy images of the localization of alphaTub protein (green) (**A1**–**A4**) and RCC1 (red) (**B1**–**B4**) during metaphase Immunostaining of HEK293T cells (*H.sapiens*). Acetone–methanol fixation.

**Figure 19:**
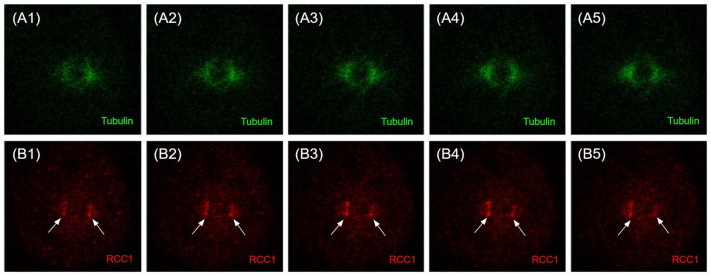
Confocal microscopy images of the localization of alphaTub protein (green) (**A1**–**A5**) and RCC1 (red) (**B1**–**B5**) during metaphase Immunostaining of U87MG cells (*H. sapiens*). Acetone–methanol fixation. The white arrows indicate the accumulation of the RCC1 signal near the centrioles.

**Figure 20:**
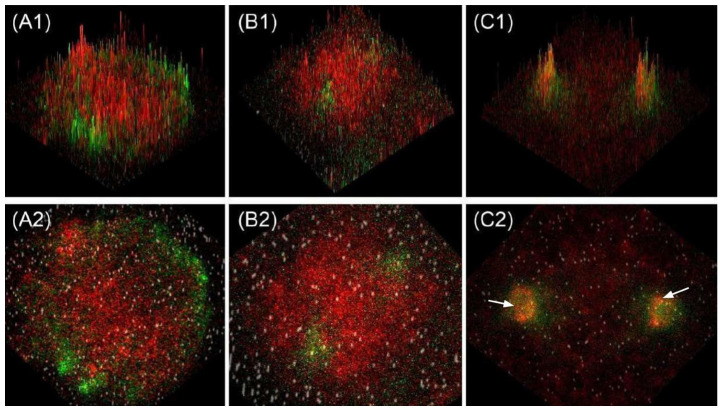
Confocal microscopy images (2.5D signal intensity plots). Alpha-tubulin and RCC1 immunostaining of (**A1**,**A2**) Kc167 cells (*D. melanogaster*, formaldehyde fixation), (**B1**,**B2**) HEK293T cells (*H. sapiens*, acetone–methanol fixation), and (**C1**,**C2**) U87MG cells (*H. sapiens*, acetone–methanol fixation). The white arrows indicate the accumulation of the RCC1 signal near the centrioles.

It was previously believed that RCC1 can affect mitotic spindle assembly only in an indirect manner by forming a proper GTP gradient. RCC1 localizes near kinetochores to ensure proper microtubule orientation. We observed RCC1 localization not only near chromosomes but also in close proximity to the centrioles of the mitotic spindle. An analysis of optical microscopy images revealed colocalization of RCC1 and tubulin in U87MG cells, being verified by Pearson correlation coefficients, while no colocalization was detected in *D. melanogaster* Kc167 cells (Figure 16). More thorough confocal microscopy examination of the cells for which this phenomenon had been observed revealed that RCC1 and tubulin always localize to the same optical sections for all the cell lines (Figure 17, Figure 18, Figure 19 and Figure S9). However, upon signal overlapping, almost no yellow color was observed in the 3D model of a U87MG cell (Figure S9) and in 2.5 D graphs for all the cell lines (Figure 20). Furthermore, Pearson correlation coefficients were insufficient for colocalization in both *Homo sapiens* and *Drosophila melanogaster* cells, although RCC1 localization near tubulin was more prominent for U87MG human glioblastoma cells. This fact suggests that proteins might not be bound directly; instead, RCC1 can be a component of the protein complex affecting centriole microtubules. We observed this distribution of RCC1 for the transition from prometaphase to metaphase. Therefore, it can be hypothesized that not only is concentration of RCC1 important as a guanine nucleotide exchange factor near chromosomes, but it is also essential for ensuring formation of centriole microtubules in U87MG glioblastoma cells. It is also possible that confocal microscopy cannot detect RCC1–tubulin colocalization because of the collateral orientation of these proteins. It is observed most vividly for the 3D model of a U87MG cell (Figure S9).

According to the Mann–Whitney U-test, the differences in RCC1 colocalization in HEK293T and U87MG cell lines are statistically significant (*p*-value = 0.00).

Optical microscopy revealed potential colocalization of RCC1 and tubulin for U87MG human cell lines, which was supported by Pearson’s correlation coefficients (Figure 16). However, a more thorough confocal microscopy study revealed that although proteins resided within the same planes and lay next to each other, calculation of the Pearson’s correlation coefficient yielded no statistically significant values (data not shown). It can indicate that tubulin and RCC1 are bound into a single protein complex, but proteins either do not interact directly or can interact within a plane impeding colocalization analysis.

### 2.4. The Role of RCC1 in Gliomagenesis

**Figure 21:**
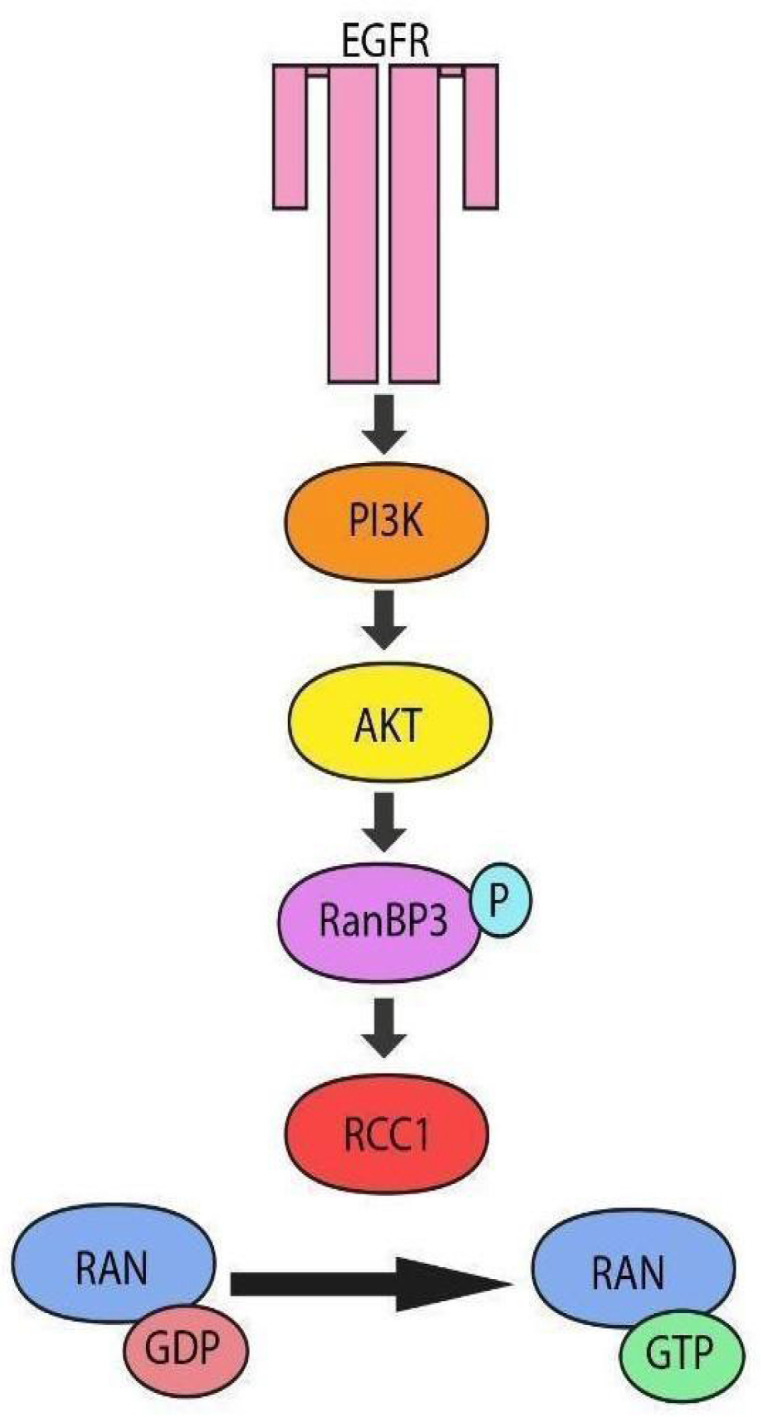
The scheme of RCC1 function regulation via the EGFR/PI3K/Akt pathway in *D. melanogaster* and *H. sapiens*.

There is a *D. melanogaster* model of glioma simulating the disease by inserting mutations in the genes encoding epidermal growth factor receptor (EGFR) and phosphoinositide 3-kinases (PI3K) [42]. These proteins form the EGFR/PI3K/Akt signaling pathway (Figure 21), one of the key pathways involved in regulation of cell proliferation, growth, differentiation, metabolism, survival, apoptosis, and angiogenesis activation. Furthermore, it participates in regulation of the RCC1 function. Interestingly, 29 proteins out of the 31 core components of this pathway in *H. sapiens* have a homolog in *D. melanogaster*.

EGFR plays a crucial role in gliomagenesis, as well as development of chemotherapy resistance in humans [43]. Its abnormal activation can disrupt the phosphatidylinositol-3-kinase (PI3K)/AKT signaling pathway, which may further alter regulation of the RCC1 function and eventually cause RCC1 malfunctioning.

**Figure 22:**
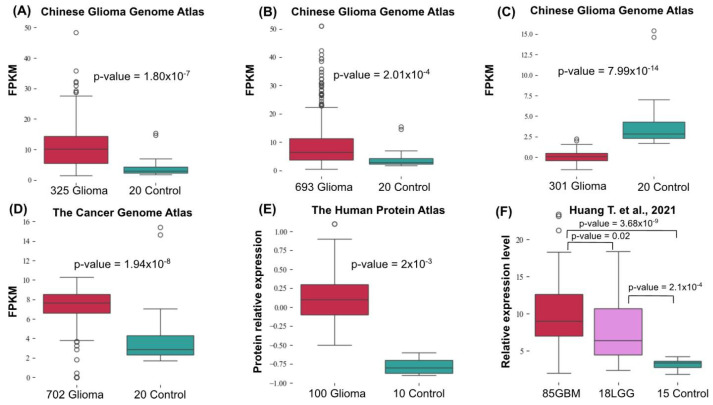
Differences in *RCC1* expression according to the Chinese Glioma Genome Atlas (CGGA). The RNA sequencing data shown in the diagrams (**A**,**B**) were obtained using an Illumina HiSeq system; the data shown in diagram (**C**) were obtained using the microarray sequencing technology. (**D**) The RNA sequencing data deposited in the Cancer Genome Atlas (TCGA). (**E**) The data on normalized protein expression (nRPX) available in the Human Protein Atlas. (**F**) The RNA sequencing data reported by Huang T. et al. (2021) [20]. The *p*-value for (**E**) was identical to that in the Human Protein Atlas.

In order to assess changes in *RCC1* expression, we analyzed the open-source data presented in the Chinese Glioma Genome Atlas, the Cancer Genome Atlas, the Human Protein Atlas, as well as the findings reported by Huang T. et al. [20] (Figure 22).

To make our own assessment, we conducted RT-PCR of ten glioma specimens and nine healthy tissue specimens obtained from patients of the Ya.L. Tsiv’yan Novosibirsk Research Institute of Traumatology and Orthopedics.

Prior to RT-PCR, six candidate reference genes (*EIF2B*, *MRPL19*, *TFRC*, *TBP*, *CTBP1*, and *B2M*) were selected based on the literature data [44] and information about commonly used genes. These candidate reference genes were tested for the following cell lines: HEK293T, HUES9, U87MG, healthy glial cell line, as well as tumor and healthy tissue specimens. Genetic stability was calculated using the RefFinder tool [45]. Three most stable reference genes (*EIF2B1*, *MRPL19*, *TBP*) were selected and used in further study.

**Figure 23:**
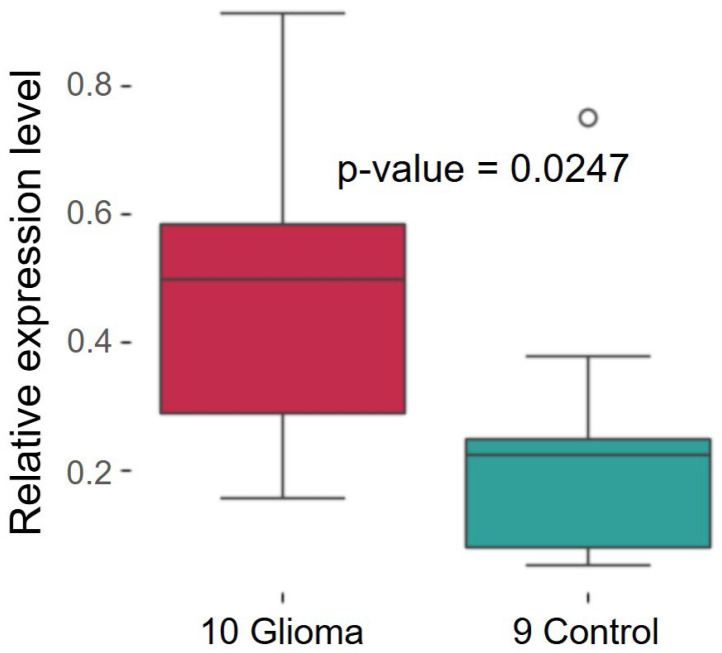
Changes in *RCC1* expression in patients with glioma.

Figure 23 demonstrates that *RCC1* expression in tumor specimens increased approximately twofold. If one takes into account differences between cell lines, the expression level in the U87MG line was increased threefold compared to that in healthy glial cell line. However, closer consideration reveals that some patients either exhibited no differences in expression or had non-significant differences. However, analysis of the data available in the Chinese Glioma Atlas [46] and the Cancer Genome Atlas reveals that statistical significance increases for larger sample size.

## 3. Materials and Methods

### 3.1. Study Objects

The *Drosophila melanogaster* fruit fly was used as a study object. Flies were reared on a standard medium consisting of cornmeal, yeast, agar, and molasses at 18, 22, or 25 °C. The Oregon-R strain was used as the wild-type control. The *Rif1^1^* strain was kindly provided by Jared Nordman (Department of Biological Sciences, Vanderbilt University, USA). The w; ru h *SuUR^ES^*, XYˆ XYˆXYˆ yw; *SuUR^ES^*, *Su(var)3-9^06^*, XYˆ XYˆXYˆ yw; *Rif1^1^*; and *SuUR^ES^*, *Su(var)3-9^06^* strains were previously obtained in our laboratory.

Cell cultures: human embryonic kidney cells HEK293T, human glioblastoma cell line U87MG, human pluripotent cells HUES9, healthy human nasal glial cell line, and *D. melanogaster* embryonic cell line Kc167s.

Tumor samples: The studies were conducted in full compliance with ethical standards developed on the basis of the Helsinki Declaration of the World Medical Association “Ethical Principles of Scientific Medical research involving humans” as amended in 2000, and the “Rules of Clinical Practice in the Russian Federation”, approved by Order of the Ministry of Health of the Russian Federation dated 1 April 2016 No. 200n. The study was approved by the Committee on Biomedical Ethics of the Novosibirsk Scientific Research Institute of Traumatology and Orthopedics named after Ya.L. Tsiv’yan” of the Ministry of Health of the Russian Federation (Protocol No. 094/15 dated 28 December 2015). All patients were individually informed about the objectives of the research and before they started, each patient provided voluntary consent, and all personal data was depersonalized.

### 3.2. RNA Extraction and Reverse Transcription

RNA was extracted from tumor and healthy tissues obtained from the Ya.L. Tsiv’yan Novosibirsk Research Institute of Traumatology and Orthopedics using QIAzol Lysis Reagent for purification of total RNA from fatty tissues (QIAGEN Sciences Inc., Germantown, MD, USA) following the manufacturer’s protocol. The extracted RNA was immediately treated with DNase I, RNAse-free (Thermo Fisher Scientific Inc., Waltham, MA, USA, # EN0525). The treated samples were immediately used in the reverse transcription reaction. Reverse transcription reactions were carried out using RevertAid reverse transcriptase (Thermo Fisher Scientific, Waltham, MA, USA) according to the manufacturer’s protocol. Obtained cDNA samples were stored at −20 °C until usage. The mRNA expression level of the gene of interest in tumor, healthy tissues and cell lines was analyzed.

### 3.3. Quantitative PCR

Quantitative PCR was conducted using BioMaster HS–qPCR SYBR Blue master mix (2×) (BiolabMix, Novosibirsk, Russia, #MHC030-2040). The following reagents were used to carry out a 25 µL reaction: 2× Master Mix—12.5 µL; 0.3 mM of forward and reverse primers; cDNA—1.3 µL; H_2_O up to 25 µL. All reactions were performed in triplicate using a LightCycler 480 Instrument II (Roche, Basel, Switzerland) according to the following program: at 95 °C for 5 min, followed by 45 cycles at 95 °C for 15 s, at the annealing temperature of 62 °C for 20 s, and at 72 °C for 30 s. The reference genes (*EIF2B1*, *MRPL19*, *TBP)* used for the normalization of the transcription levels of the *RCC1* gene were selected in this study. The primers used in this study are listed in the Table S2 (Supplementary Materials). The primers for amplification of the *RCC1* transcripts were designed using Primer-BLAST NCBI [47]. Primers for *RCC1* were selected to target a region conserved across all forms of human RCC1. The primers for RCC1 used in this study were as follows:

RCC1_F: AGCTGCAAGAGAAGGTGGTA
RCC1_R: ACACCGTTATTGTCCCGGAAG

External standard curves for each gene were constructed to determine primer efficiencies. The relative quantification was carried out using the “Relative quantification analysis with high confidence 2nd derivative max method” supplied with the LightCycler 480 software.

### 3.4. Cultivation and Cryopreservation

Cells were cultured in a CO_2_ incubator at 37 °C and 5% CO_2_ content. The growth medium consisted of MEM or DMEM supplemented with 15% fetal bovine serum and a combination of antibiotics: streptomycin (100 µg/mL), penicillin (100 µg/mL), and amphotericin B (2.5 µg/mL). Cell culture passage was performed after a dense monolayer of cells had been formed, typically two or three times per week; the split ratio was 1/2–1/4. The cell culture passage procedure was performed as follows: the growth medium was removed; cells were rinsed twice with DPBS solution, and 0.5–1 mL of a trypsin–EDTA mixture (trypsin, 2.5 mg/mL; EDTA, 0.3 mg/mL) was added. The cells were incubated at 37 °C for 2–5 min for monolayer destruction. Then, 5 mL of growth medium was added; the cells were resuspended and transferred to new appropriately sized culture flasks containing a sufficient amount of fresh growth medium for subsequent cultivation. Actively growing cells were used for cryopreservation (freezing). Prior to freezing, cells were processed using the same procedure as the one utilized for routine cell culture passage, but at the end, the cell suspension was transferred into a 15 mL tube and centrifuged at 2000 rpm (600× *g*) for 5 min. The pellet was resuspended in a solution containing 90% newborn calf serum and 10% DMSO. The cell suspension was aliquoted into plastic cryovials, placed into cryo-containers, and exposed to −70 °C for at least 24 h. The ampoules were then transferred to liquid nitrogen for long-term storage.

### 3.5. Immunostaining of Polytene Chromosomes

The slides were rinsed with PBST solution for immunostaining. Without letting the slide dry out, primary antibodies in blocking buffer (2% bovine serum albumin in PBST) were added to the specimens, followed by 2 h incubation in a humid cabinet at room temperature. The slides were then washed with a PBST solution, and a solution of fluorochrome-conjugated secondary antibodies in blocking buffer was added. The slides were incubated for 1.5 h, and the slides were washed with PBST buffer once again (three times, 5 min each). Next, 8 µL of a solution (a DABCO–DAPI mixture, Abcam (Cambridge, UK)) was placed dropwise onto the dried specimens, and a coverslip was placed on top. An Olympus BX-50F microscope (Olympus, Tokyo, Japan) was used for cytological analysis and image recording. Each experiment was repeated at least five times; the staining pattern was reproducible in all cases. Antibodies for *Drosophila* RCC1 and NUP93-1 were provided by N.E. Vorobyeva, Group of transcriptional complexes dynamics, Institute of Gene Biology RAS.

### 3.6. Immunostaining of Cells with Formaldehyde Fixation

Cells in PBS solution were centrifuged at 800× *g* for 5 min. The fluid was removed; 1 mL of PBS was added to the pellet to wash cells, and centrifugation was repeated. The supernatant was removed, and the cells were suspended in a small amount of the remaining solution. Formaldehyde (3.7% in PBS; 2 mL) was added dropwise. The mixture was shaken carefully and incubated at room temperature for 7 min. After centrifugation at 800× *g* for 5 min, the supernatant was removed, and 2 mL of PBS was added to dilute the remaining formaldehyde. The mixture was centrifuged at 800× *g* for 5 min. The supernatant was removed, and 0.5–1 mL of PBS was added. Fixed cells were loaded into Cytospin funnels (100 µL per funnel) and centrifuged at 900 rpm for 4 min. Microscope glass slides were submerged into liquid nitrogen for 3–5 min. The glass slides were transferred into PBS until all of them were ready for immunostaining. The glass slides were incubated in PBS + 0.1% Triton (PBT) for 30 min, followed by 30 min incubation in PBS + 3% BSA. Twenty-five milliliters of primary antibody solution in PBS + 3% BSA were applied. The glass slides were covered with parafilm and incubated overnight at 4 °C. After primary staining, the glass slides were washed first with PBS for 10 min, then with PBT for 20 min, and finally with PBS for 5 min. Twenty-five microliters of secondary antibodies in PBS + 3% BSA solution were added. The glass slides were covered with parafilm and incubated at room temperature for 2 h. Next, the glass slides were washed again with PBS for 10 min, with PBT for 20 min, and with PBS for 5 min. DAPI-containing antifade mounting medium (5 µL) was applied, and coverslips were placed over the glass slides. The resulting specimens were stored in a dark place. An Olympus BX-50F microscope (Olympus, Tokyo, Japan) and an LSM 700-LK 2 laser confocal microscope (Zeiss, Oberkochen, Germany) were used for cytological analysis and image recording.

### 3.7. FISH

Salivary glands were dissected in Ephrussi-Beadle solution and then fixed in a 3:1 mixture of ethanol and acetic acid for 30 min at −20 °C, squashed in 45% acetic acid, snap-frozen in liquid nitrogen and stored in 70% ethanol at −20 °C. Fluorescence in situ hybridization (FISH) on polytene chromosomes was performed as described [11]. Random-primed labeling of DNA probes with TAMRA (TMR, Tetramethylrhodamine) was done using Klenow enzyme.

### 3.8. Immunostaining with Acetone–Methanol Fixation

HEK293T cells were cultured in the DMEM growth medium (Gibco, Loughborough, UK) supplemented with 10% FBS (Gibco, UK) and 1× Penicillin–Streptomycin (HyClone, Logan, UT, USA) at 37 °C in an atmosphere of 5% CO_2_. A round coverslip (25 mm in diameter) pretreated with ethanol was placed in a Petri dish (35 mm), and 5–6 × 10^5^ cells in 2 mL of growth medium was transferred. The cells were incubated at 37 °C for 24 h in an atmosphere of 5% CO_2_. The growth medium was then removed from the Petri dish; the cells were washed twice with 1 mL of PBS solution (room temperature). The cells were fixed on ice using cold methanol–acetone solution for 20 min and washed with PBS solution thrice (each washing cycle lasting 10 min). The cells were then co-incubated with blocking buffer for 30 min and then with incubation buffer containing the primary antibody for 2 days at 4 °C. Next, the mixture was washed with PBS solution thrice (each washing cycle lasting 10 min) and incubated in the buffer containing secondary antibody at room temperature for 2 h. The cells were also washed with PBS thrice (each washing cycle lasting 10 min). DAPI-containing antifade mounting medium (5 µL) was applied, and the slide with a cell culture was flipped onto a glass slide. An Olympus BX-50F microscope and an LSM 700-LK 2 laser confocal microscope were used for cytological analysis and image recording.

### 3.9. Western Blot Hybridization

For Western blot hybridization, *Drosophila* salivary gland tissue (20 pairs per lane) and human HEK cell culture (approximately 10^5^ cells per lane) were used. All samples were lysed in a buffer (50 mM Tris-HCl, 150 mM NaCl, 0.1% Triton X-100, 0.1% SDS, and 1 mM PMSF), and the total protein extract was loaded onto a polyacrylamide gel. The proteins in the samples were separated electrophoretically in a Bio-Rad chamber using a standard protocol on a 10% SDS-polyacrylamide gel. After separation, a membrane was placed on top of the gel, and electrical transfer of the sample was carried out using a cooled buffer solution (0.2 M glycine, 0.025 M Tris, and 20% methanol) for 1 h at a direct current. Primary antibodies were diluted in blocking solution (rabbit polyclonal antibodies against Xenopus RCC1 protein from Invitrogen—1:1000; mouse monoclonal antibodies against histone H3 from Novus Biological—1:10,000). The membrane was placed in diluted primary bodies and incubated for 1 h at room temperature on a shaker at 45–50 rpm. After that, it was washed with 1× PBST. Immunodetection was then performed using the Novex ECL Chemiluminescent Substrate Reagent Kit (Invitrogen, Carlsbad, CA, USA), and the results were analyzed using the Amersham Imager 600 topographer (GE Healthcare Life Sciences, Chicago, IL, USA) and the ImageJ 1.54.P program [48].

### 3.10. Bioinformatics Analysis

The open-source data of the Chinese Glioma Atlas, the Cancer Genome Atlas, the Human Protein Atlas, as well as the findings reported by Huang T. et al. (2021) [20] were used to analyze *RCC1* expression.

Statistical analysis of the Mann–Whitney U test was conducted using the scipy.stats library.

The interactions were analyzed using open-source data from the STRING database and the Python Network X library.

## 4. Conclusions

The study demonstrates a high degree of evolutionary conservation of the RCC1 protein between *Drosophila melanogaster* and *Homo sapiens*. This is manifested by the preservation of key functional domains and the ability to form similar protein complexes.

In U87MG glioblastoma cells, the RCC1 protein is localized near centrioles during mitosis. Combined with data on the increased expression of the *RCC1* gene in gliomas, this suggests that a disruption of its normal localization and level of expression may be associated with mechanisms of oncogenesis.

The results obtained expand our understanding of RCC1’s functions beyond regulation of chromatin condensation, indicating its potential role in glioblastoma pathogenesis. This opens up new avenues for further investigation of this gene as a potential therapeutic target.

## Figures and Tables

**Table 1:** Comparison of the organization of the *RCC1* gene in *Homo sapiens* and *Drosophila melanogaster*.

	*Homo sapiens*	*Drosophila melanogaster*
Gene positions	chr1: 28 506 043-28 538 989 GRCh38/hg38 chr1:28 529 867-28 538 007	chr3L:5 929 674-5 933 200 Release 6.54
Number of transcripts	6	3
Size	32,947 bp (including UTRs)	2351 bp
Exons	10–13 Number of coding exons: 9 Total exon length: 1266 bp	Total number of exons: 3–4 Total exon length: 2017 bp
Introns	Number of introns: 9–11 Total intron length: 29,125	Number of introns: 2–3 Total intron length: 207 bp
Protein	bpNP_001041659.1 (isoform a): 452 aa NP_001041660.1 (isoform b): 438 aa NP_001260.1 (isoform c): 421 aa 45 kDa [1,7]	NP_523943.1 (isoform a): 547 aa NP_729118.1 (isoform b): 547 aa NP_729119.1 (isoform c): 547 aa 58.9 kDa [7], 68 kDa [8,9]

**Table 2:** Functional annotation of gene groups for *Drosophila melanogaster*.

No.	Group Members	Functions of the Group
1	RanGAP, Nup358, Rcc1, Ran	Nucleocytoplasmic transport, chromatin condensation, proper mitotic progression
2	Nup107, Nup44A, Nup154, Nup160, Nup153, Elys	Nuclear pore components, double-strand break repair, involvement in regulation of female and male fertility, association with transcriptionally active chromatin
3	HDAC1, Caf1-55, Jarid2, Pc, Pcl, E(z), Su(z)12, esc, escl, ph-p, Psc, Sce, ph-d, Kdm2	Polycomb repressive complex, syncytial blastoderm mitotic cell cycle. Chromatin regulators, transcription regulators
4	Su(var)3-7, HP4, HP6, LBR, Lhr, Lam, Su(var)205, HP1c, Su(var)3-9, His1	Pericentric heterochromatin, protein binding, nucleus, heterochromatin, telomeric region of a chromosome
5	Su(var)3-7, Chro, BEAF-32, Su(Hw), Cp190	Protein binding, binding of chromatin insulator sequence, DNA binding, zinc fingers
6	SuUR, Cdc45, Rif1	Control of chromatin organization in polytene chromosomes
7	piwi, aurB	Mediation of both meiotic and mitotic chromosome segregation; repression of mobile elements during meiosis through the formation of PIWI/piRNA protein complexes; and regulation of methylation and subsequent repression of transposons
8	His 3: CG31613, His 3.3A, His 2: CG31618	Histone proteins

**Table 3:** Functional annotation of gene groups for *Homo sapiens*.

No.	Group Members	Functions of the Group
1	RCC1, Ran, RANBP1, KPNB1, RANBP2, RANGAP1	Nucleocytoplasmic transport, chromatin condensation, and proper mitotic progression
2	NUP205, NUP37, NUP155, NUP35, NUP160, NUP93, NUP43, NUP214, NUP133, NUP107, AHCTF1, NUP98, NUP85, SEC13, SEH1L	Components of the Nup107-160 subcomplex of the nuclear pore complex (NPC). The Nup107-160 subcomplex is essential for the assembly of a functional NPC. Furthermore, the Nup107-160 subcomplex is needed for proper kinetochore-microtubule attachment, mitotic progression, and chromosome segregation
3	EZH2, EED, SUZ12, YAF2, RBBP7, YARID2, RBBP4, PCGF1, CBX7, RNF2, BMI1, COMMD3-BMI1, RING, PHC1, CBX8, CBX2, PCGF1, PCGF2, PHC2, CBX4	Polycomb repressive complex. Chromatin regulators, transcription regulators
4	UBE2I, SUMO1, SUMO2	Protein SUMOylating
5	SUV39H1, ATM, RIF1, H4C6, CBX1	Telomeric region of a chromosome, DNA damage response

## Data Availability

The original contributions presented in this study are included in the article/Supplementary Materials. Further inquiries can be directed to the corresponding author.

## Supplementary Materials

The following supporting information can be downloaded at: https://www.mdpi.com/article/10.3390/ijms262311276/s1.
