# Supplementary material for: The Gene Encoding the RCC1 (Regulator of Chromosome Condensation 1) Protein in Drosophila melanogaster and Homo sapiens
Source: Int J Mol Sci. 2025 Nov 21;26(23):11276. doi: 10.3390/ijms262311276 (PMC12691783; doi:10.3390/ijms262311276)
Supplement: Supplementary file 1 [file ijms-26-11276-s001.zip › ijms-3888060-supplementary.pdf]

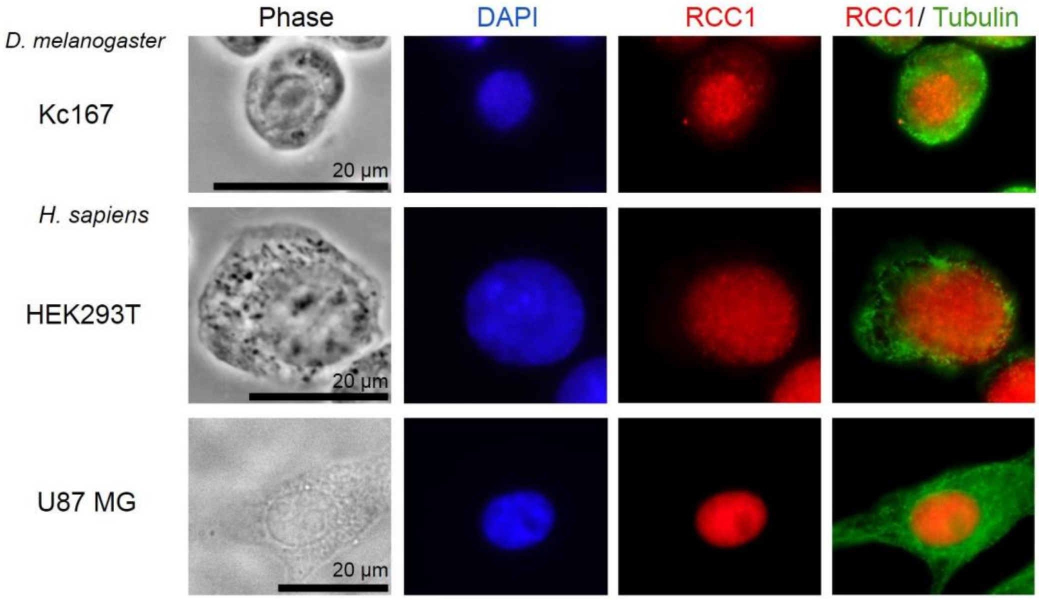

Figure S1. Optical microscopy images of the localization of RCC1 (red) and alphaTub (green) proteins during interphase. Immunostaining of Kc167 cells (*D. melanogaster*) and HEK293T, U87MG cells (*H. sapiens*). Fixation with 3.7% formaldehyde.

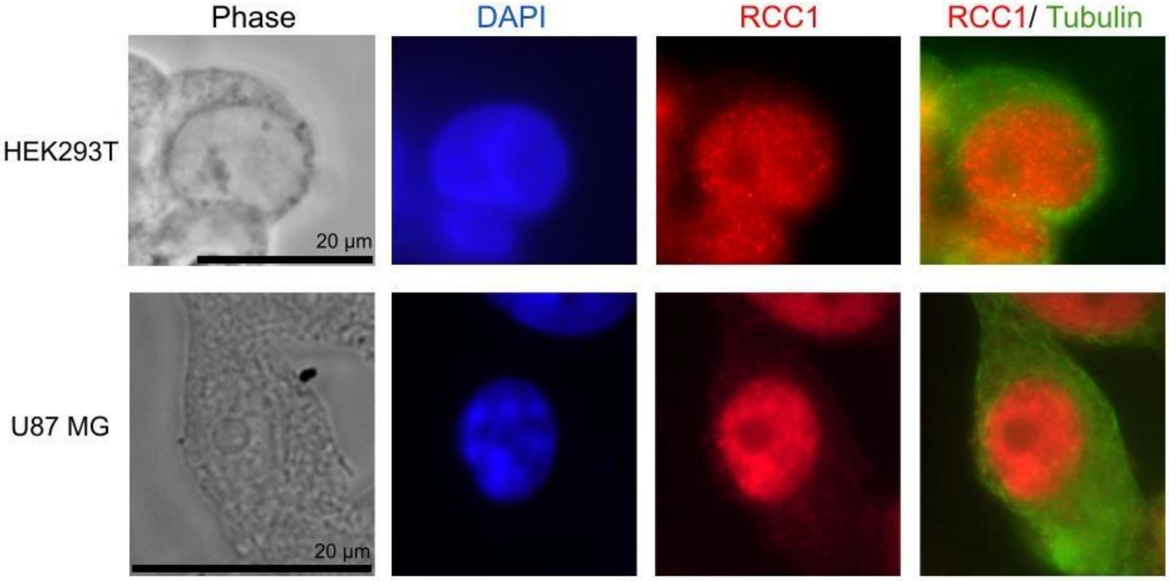

Figure S2. Optical microscopy images of the localization of RCC1 (red) and alphaTub (green) proteins during interphase. Immunostaining of HEK293T and U87MG cells (*H. sapiens*). Acetone–methanol fixation

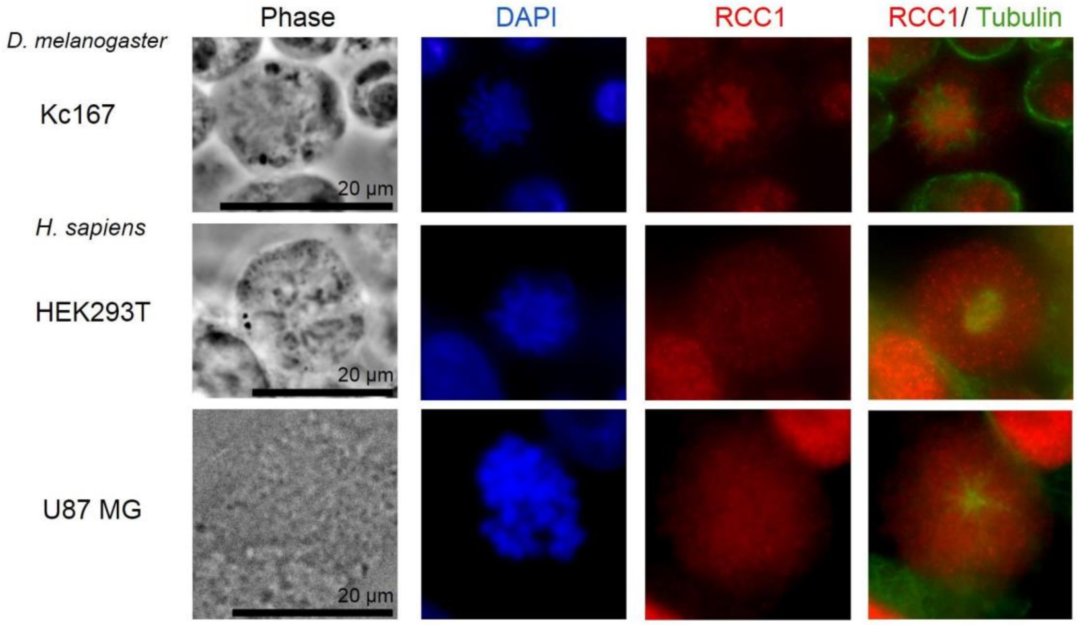

Figure S3. Optical microscopy images of the localization of RCC1 (red) and alphaTub (green) proteins during prophase. Immunostaining of Kc167 (*D. melanogaster*), HEK293T, and U87MG (*H. sapiens*) cells. Fixation with 3.7% formaldehyde.

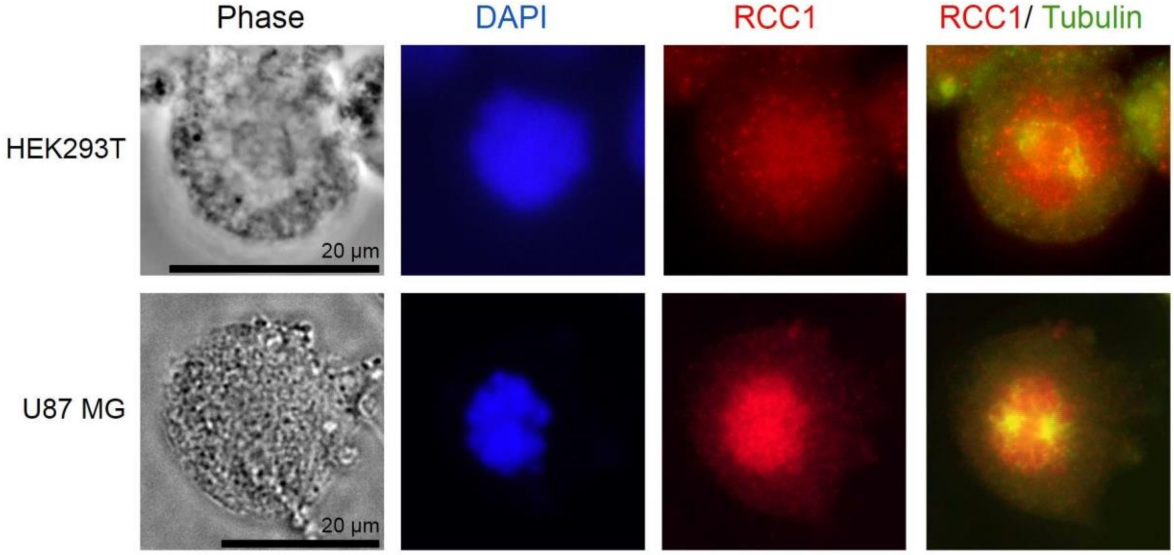

Figure S4. Optical microscopy images of the localization of RCC1 (red) and alphaTub (green) proteins during prophase. Immunostaining of HEK293T and U87MG cells (*H. sapiens*). Acetone–methanol fixation.

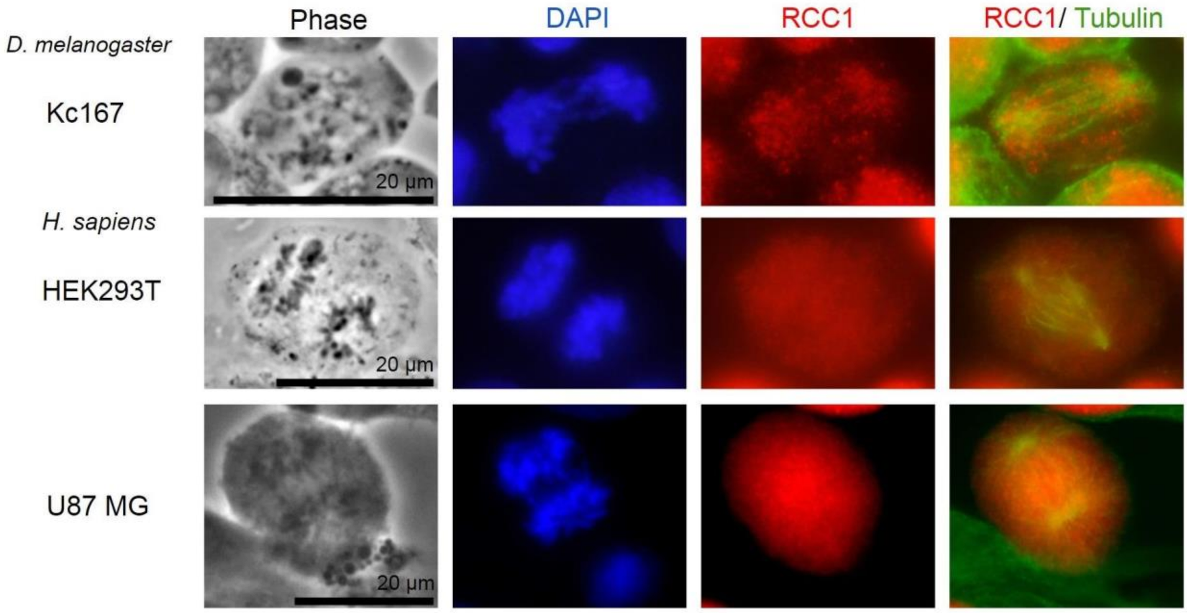

Figure S5. Localization of RCC1 (red) and alphaTub (green) proteins during anaphase. Immunostaining of Kc167 (*D. melanogaster*), HEK293T, and U87MG cells (*H. sapiens*). Fixation with 3.7% formaldehyde.

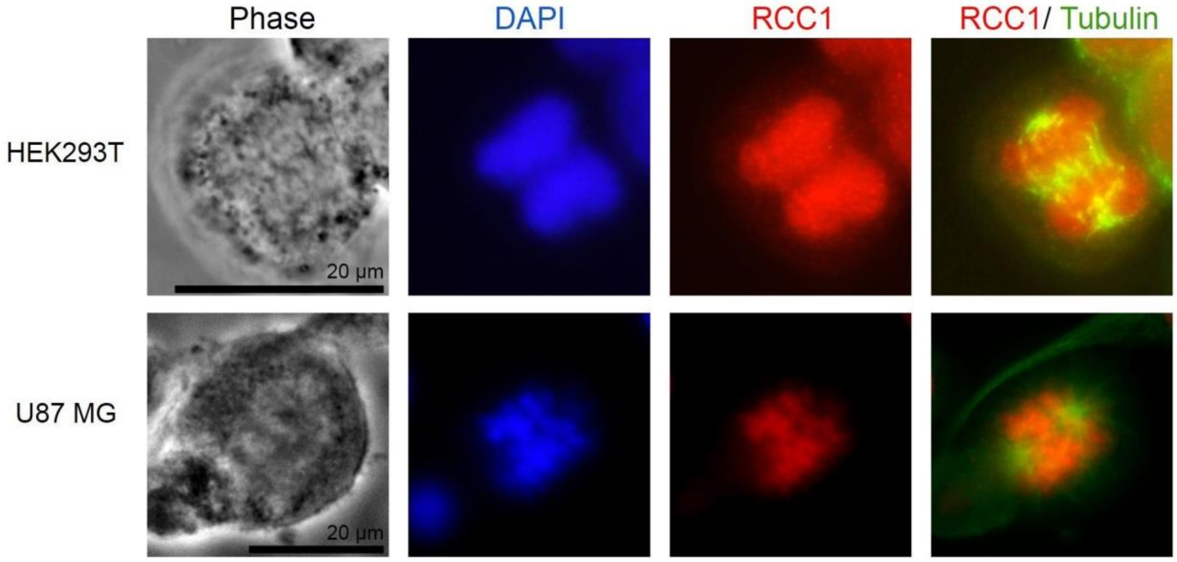

Figure S6. Localization of RCC1 (red) and alphaTub (green) proteins during anaphase. Immunostaining of HEK293T and U87MG cells (*H. sapiens*). Acetone–methanol fixation.

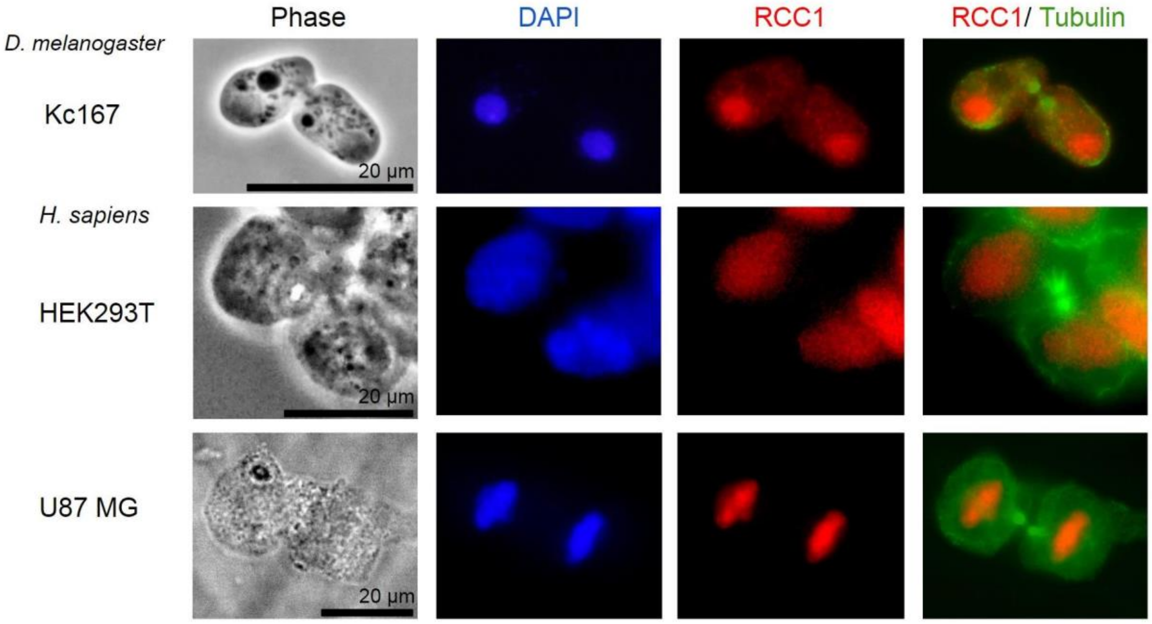

Figure S7. Localization of RCC1 (red) and alphaTub (green) proteins during telophase. Immunostaining of Kc167 (*D. melanogaster*), HEK293T and U87MG cells (*H. sapiens*). Fixation with 3.7% formaldehyde.

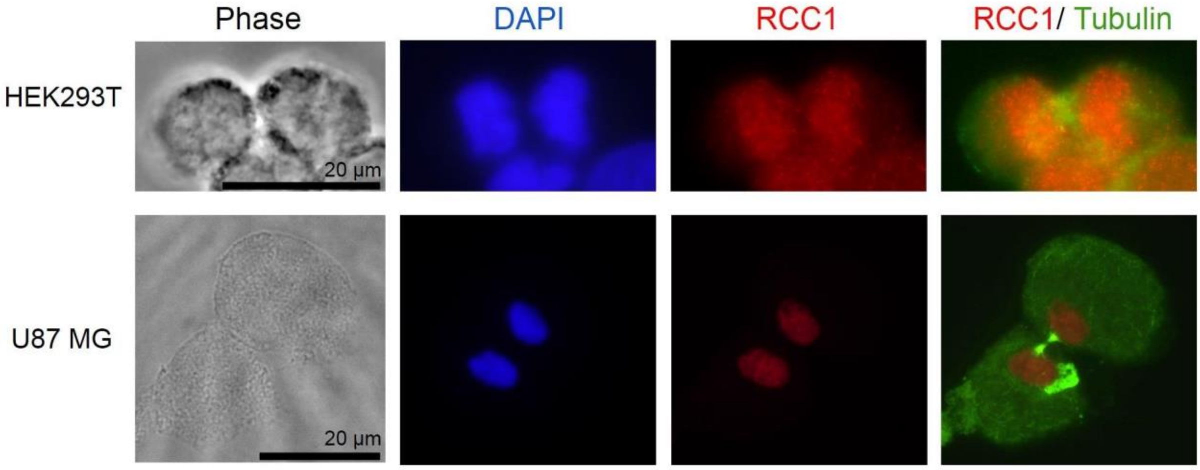

Figure S8. Localization of RCC1 (red) and alphaTub (green) proteins during telophase. Immunostaining of HEK293T and U87MG cells (*H. sapiens*). Acetone–methanol fixation.

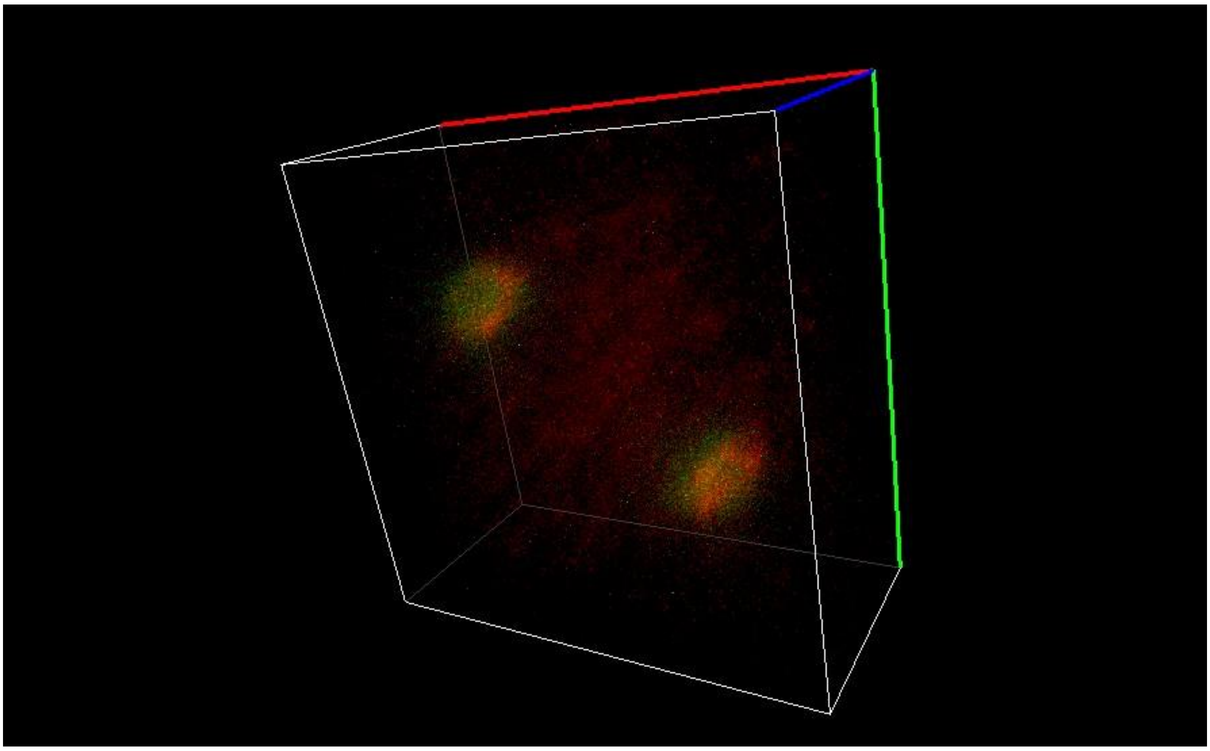

Figure S9. A 3D confocal microscopy image of a U87MG cell (*H. sapiens*) obtained by superposition of z-stacks. Alpha-tubulin and RCC1 immunostaining. Acetone–methanol fixation.

Table S1. Localization of anti-RCC1 antibodies in polytene chromosomes of the *Rif1*[1] and *SuUR_ES, Rif11,Su(var)3-9** No data are available for this strain (three mutations) for chromosomes 4 and 3R.

<table>
<tr><th>Chr 4 (Rif1¹)</th><th>Chr X (Rif1¹)</th><th>Chr X (SuUR_ES, Rif1¹, Su(var)3-9)</th><th>Chr 2L (Rif1¹)</th><th>Chr 2L (SuUR_ES, Rif1¹, Su(var)3-9)</th><th>Chr 2R (Rif1¹)</th><th>Chr 2R (SuUR_ES, Rif1¹, Su(var)3-9)</th><th>Chr 3L (Rif1¹)</th><th>Chr 3L (SuUR_ES, Rif1¹, Su(var)3-9)</th><th>Chr 3R (Rif1¹)</th></tr>
<tr><td>101DF</td><td>1B1-4</td><td>1B</td><td colspan="2">21D1-2</td><td colspan="2">41AE</td><td>61C1-2</td><td>61C</td><td>82EF</td></tr>
<tr><td>102D</td><td>1E1-4</td><td>1EF</td><td colspan="2">21E1-2</td><td>41F1-3</td><td></td><td colspan="2">61F1-4</td><td>83D1-5</td></tr>
<tr><td></td><td></td><td></td><td colspan="2">22A1-3</td><td colspan="2">42A1-2</td><td>62A1-2</td><td></td><td>84A1-2</td></tr>
<tr><td></td><td colspan="2">3A1-4</td><td colspan="2">22B1-2</td><td colspan="2">42B1-3</td><td>62B1-2</td><td></td><td>84A4-5</td></tr>
<tr><td></td><td>3C1-6</td><td></td><td>22C1-2</td><td></td><td>43A1-2</td><td>43A</td><td>62C1-2</td><td></td><td>84B1-2</td></tr>
<tr><td></td><td>3DE</td><td></td><td></td><td>22CD</td><td>43B1-2</td><td>43BC</td><td>62D1-2</td><td></td><td>84D1-4</td></tr>
<tr><td></td><td colspan="2">4A1-2</td><td>22F</td><td></td><td colspan="2">44CD</td><td colspan="2">63A1-3</td><td>84E1-2</td></tr>
<tr><td></td><td>4B1-4</td><td>4B1-2</td><td colspan="2">23A</td><td>44F1-3</td><td>44F1-2</td><td colspan="2">63E1-6</td><td>84EF</td></tr>
<tr><td></td><td>4C1-4</td><td>4C1-2</td><td></td><td>23B</td><td colspan="2">45A1-2</td><td>64C1-5</td><td></td><td>85A1-6</td></tr>
<tr><td></td><td colspan="2">4D1-2</td><td></td><td>23C</td><td>45C1-4</td><td>45C1-2</td><td></td><td>64C4-5</td><td>86A1-8</td></tr>
<tr><td></td><td>4E1-2</td><td></td><td>23E1-2</td><td>23E</td><td colspan="2">45D1-2</td><td></td><td>64D1-2</td><td>86B1-2</td></tr>
<tr><td></td><td>4F1-4</td><td></td><td></td><td>24A</td><td colspan="2">45E1-2</td><td colspan="2">64D1-6</td><td>86C1-2</td></tr>
</table>

5A1-8		5A		24D1-2		46A1-2		46A1-4		64E1-2		86D1-5
		5C	24E1-2				47A1-2			64F1-2		86E1-8
	5D1-6				24F	47B1-7		47B	65A1-6		65A	87B1-5
	6A1-2			25A1-4		47D1-2		47D1-4	65B1-2		65B	87C1-2
	7B1-2		25E1-2		25E	48AB			65C1-2			87D1-5
7C1-2			25F1-2		25F			48A1-4	65D1-3		65D	87E1-2
	7E1-2		26A1-2		26A		48C1-2		65E1-4		65E	87F1-2
8A1-2		8AB	26CD		26C1-2		48D1-2		65E7-9			88A1-2
8B1-4					26D		48E1-2			65F		88B1-2
	8C1-2		26EF					49A1-2	66A1-2		66A-C	88E1-4
		8E1-4		27A				49D1-3	66A5-9			89A1-4
	9A1-4				28A		49F1-2				66E	89D1-2
9E1-3				28D			50A1-4			67A1-4		89E1-4
	10A1-2		29D1-2		29DE		50C1-4				67C	90A1-2
	10B1-2		29EF			51A1-5		51A	67D9-13			90B1-3
	11A6-9				29F	51C1-2		51C1-4			67D9-10	90DE
11C		11C1-2	30A1-2		30A	51D1-2			67F1-2			91B1-4
11D		11D1-3		30B		51E1-2			68A1-2		68A	91D1-2
	12A1-2				30C			52A		68E1-4		92A1-2
	12D1-2				30D			52C		69D1-2		92B1-2
13A1-2		13A			30E	53A1-2				70A1-5		92C1-2
13B1-4		13B		31A1-2		53B1-2		53BC		70C1-2		92D1-2
13C1-2					31B1-2		54AB			70DE		92E1-2
13D1-2					31C	55A1-4				71A1-2		chr 3R
		13E1-2			31D			55AB		71C1-2		92F1-2
14A1-2		14AB	32A1-2			55C1-5		55C		71F1-2		93A1-5
14B1-4				32B1-2		56AB			72A1-2			93B1-3
14D1-2			32C1-2					56D			72DE	93E1-4
15B1-2			32E1-2		32E	56F1-10		56F1-7	72E1-2			93F9-10
15D1-2			32F1-2		32F		57A1-4			73A1-4		94A1-5
16A1-9		16A		33A1-2			57B1-6			74A1-6		94D1-4
16D1-2					33B1-2		58A1-4		75A1-2			95A1-10
16F1-4			33CD			59A1-3		59A		75C1-2		96A1-2
17A1-5					33D1-4	59C1-2		59C		76A1-4		96C1-2

17B1-2		33F1-2		33F		59D1-4		76EF		96D1-2
17C1-3	17CD	34A1-2		34A1-4		60CD		77A		96E1-2
17D1-2				34D	60E				77E1-4	97A1-6
18A1-3	18A1-2		34EF		60F1-3		60F2-3		78A1-2	97B1-4
18C1-4	18C		35AB					79D1-2		98A1-3
19A1-4	19A		35C						79E1-4	98B1-2
19C1-2		35D1-4		35D					80AC	98C1-2
19E1-4		35E1-2		35E						98D1-2
		35F								98E1-2
				36A						99A1-2
				36CD						99B1-4
		36C1-2								99E1-3
		36D1-2								99F1-2
		36E1-4		36E						100A1-2
		37A1-2		37A						100B1-2
		37D1-2		37D						100B4-5
		38A1-2		38A						100C1-5
		38C1-2		38C						100F1-2
		38E1-2								∑ 63
		39B1-2		39B						92%
			39DE							
		40A1-2		40A						
		40C1-2								
				40AF						

Table S2. Primers used for RT-RCR.

**Primers**
RCC1_F: AGCTGCAAGAGAAGGTGGTA
RCC1_R: ACACCGTTATTGTCCCGGAAG
B2M_F: CACCCCCACTGAAAAAGATG
B2M_R: ATATTAAAAAGCAAGCAAGCAGAA
TFRC_F: GTCGCTGGTCAGTTCGTGATT
TFRC_R: AGCAGTTGGCTGTTGTACCTCTC
EIF2B1 _F: CTACTCCAGAGTGGTCCTGAGA
EIF2B1 _R: GTTGAGGTGGCAGAGGGCTTTG
MRPL19 _F: CAGGAAGAGGACTTGGAGCTAC
MRPL19 _R: GCTATCATCCAGCCGTTTCTCTA
CTBP1 _F: TGAGCAGCAAGCTCAGTCCAGA
CTBP1_R: TCCGTTCTCAGTTGCCTGTGGA
TBP_F: TGTATCCACAGTGAATCTTGGTTG
TBP_R: GGTTCGTGGCTCTCTTATCCTC

Table S3 Fixation with 3.8% formaldehyde

	Kc167	HEK293T	U87MG
Prophase	7	78	66
Metaphase	27	13	199
Anaphase	11	38	23
Telophase	14	12	155

Table S4 Fixation with acetone and methanol

	HEK293T	U87MG
Prophase	29	56
Metaphase	26	132
Anaphase	14	10
Telophase	15	124
